# Toxicological assessment of *Chlorella vulgaris* and its potential preventive effect in a chronic obstructive pulmonary disease (COPD) mouse model

**DOI:** 10.3389/ftox.2025.1654583

**Published:** 2025-11-11

**Authors:** Lucia Giambastiani, Sofia Fiorentino, Kylie Brady, Andrea Raffaelli, Emilia Bramanti, Erna Cecilia Lorenzini, Vincenzo Longo, Francesca Sparvoli, Luisa Pozzo, Andrea Vornoli

**Affiliations:** 1 CNR-IBBA, Institute of Agricultural Biology and Biotechnology, National Research Council, Pisa, Italy; 2 Crop Science Research Center, Scuola Superiore Sant’Anna, Pisa, Italy; 3 CNR-ICCOM, Institute of Chemistry of Organometallic Compounds, National Research Council, Pisa, Italy; 4 Department of Biomedical Sciences for Health, University of Milan, Milano, Italy; 5 CNR-IBBA, Institute of Agricultural Biology and Biotechnology, National Research Council, Milano, Italy

**Keywords:** *Chlorella vulgaris*, green microalgae, carotenoids, polyphenols, drug metabolism, inflammation, oxidative stress

## Abstract

Green microalgae, particularly *Chlorella vulgaris*, are a rich source of bioactive and nutritional compounds, making them promising candidates for nutraceutical applications. This study evaluated the antioxidant capacity, phenolic composition, and potential health effects of *Chlorella vulgaris* supplementation (1% and 8%) in male BALB/c mice over 4 weeks, as well as its preventive role in a murine model of chronic obstructive pulmonary disease (COPD). The extract showed a high antioxidant potential, supported by its phenolic and carotenoid profile. Supplementation, especially at 8%, enhanced antioxidant defences without signs of liver or kidney toxicity. In the COPD model, *C. vulgaris* reduced inflammation, improved oxidative stress balance, and partially restored normal lung structure. Additionally, changes in caecal metabolites suggested a positive impact on gut microbiota and metabolic homeostasis. Overall, *C. vulgaris* supplementation demonstrated detoxifying, antioxidant, and anti-inflammatory benefits, supporting its potential use as a functional food, particularly under stress-related conditions such as COPD.

## Graphical Abstract

**Figure:**
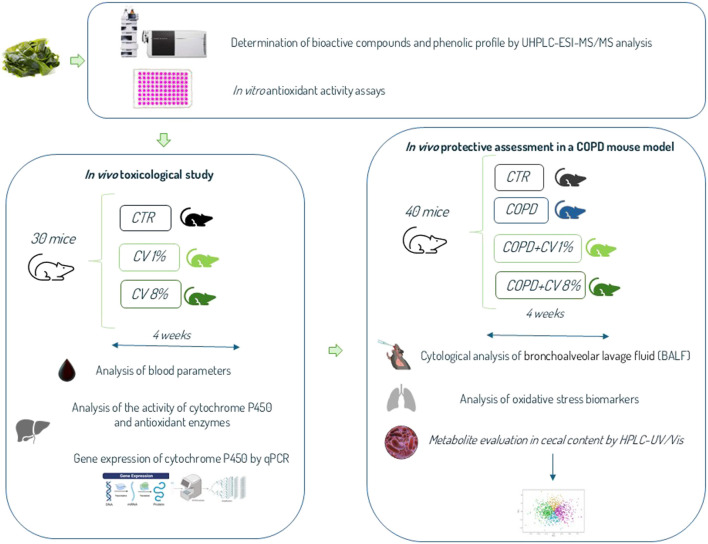

## 1 Introduction

In recent years, scientific research has increasingly focused on identifying sustainable food sources to address the growing global demand caused by population growth, climate change, depletion of natural resources, and other environmental pressures while also exploring their potential to attenuate oxidative stress and inflammatory responses (Pörtner et al., 2022; Mehariya et al., 2021). Sustainable food resources not only improve dietary habits and human health but also support economic growth and reduce environmental impact (Ferdouse, 2018; Food and Agriculture Organization of the United Nations, International Fund for Agricultural Development, United Nations Children’s Fund, World Food Programme, and World Health Organization, 2025).

Among the potential candidates, microalgae have emerged as particularly promising due to their high nutrient content and low environmental footprint. These single-celled organisms grow efficiently under diverse climatic conditions and aquatic habitats, converting CO_2_ into biomass via photosynthesis and accumulating macronutrients essential for human health.

Microalgae are especially rich in proteins comparable to animal-derived foods and contain a wide variety of secondary metabolites. These metabolites, which vary by species, represent adaptive survival mechanisms triggered by biotic and abiotic factors such as light, temperature, salinity, pH, mineral content, CO_2_ availability, and growth stage (Batista et al., 2013; Barkia et al., 2019). Moreover, microalgae are a significant source of bioactive compounds with potential therapeutic applications, including dietary fiber, polyphenols, prebiotic polysaccharides, vitamins, essential minerals, sterols, and polyunsaturated fatty acids (PUFAs), such as eicosapentaenoic acid (EPA) and docosahexaenoic acid (DHA) (Chen et al., 2023; Raposo et al., 2013).

Within this group, *C. vulgaris* has gained particular attention due to its well-balanced nutritional composition, ease of cultivation, and high biomass yield. Although native to Japan and Taiwan, *Chlorella vulgaris* is now cultivated worldwide. Its exceptional nutrient profile has led to its designation as a “nutrient-dense” food and research on its health-promoting properties began in the early 1950s.

However, despite the growing interest in its pharmacological potential, the specific bioactive compounds responsible for the beneficial effects of *C. vulgaris* remain insufficiently characterized. This limitation is partly attributed to synergistic interactions among the various nutrients and antioxidant molecules present in the biomass. Several studies have evaluated the potential of *C. vulgaris* supplementation in preventing pathological conditions in both rodent models and human trials (Bito et al., 2020), reporting its capacity to mitigate metabolic disturbances associated with obesity and diabetes (Vecina et al., 2014; Ramos-Romero et al., 2021).

The high content of macronutrients and bioactive compounds makes *C. vulgaris* an excellent candidate for nutraceutical and pharmaceutical applications, capable of promoting human health and preventing certain diseases (Abdel-Karim et al., 2019; Mendes et al., 2024). For instance, *C. vulgaris* extract has been shown to *in vitro* downregulate pro-inflammatory cytokines such as tumor necrosis factor-alpha (TNF-α), interleukin-6 (IL-6), and interleukin-1 (IL-1), thereby mitigating inflammation. Additionally, it can inhibit the production of nitric oxide (NO), which, when excessively produced by immune cells, contributes to tissue damage (Sibi and Rabina, 2016).

Inflammation is the body’s natural defense mechanism against harmful stimuli and involves both macroscopic and microscopic changes, such as redness, swelling, and immune cell infiltration (Aghasafari et al., 2019). It can be classified into acute and chronic forms: acute inflammation facilitates healing and primarily involves neutrophils, while chronic inflammation persists over time, leading to tissue remodeling and fibrosis (Moldoveanu et al., 2009; Aghasafari et al., 2019).

Among the organs most susceptible to inflammation and oxidative stress are the lungs, due to their constant exposure to inhaled agents, oxygen-rich environment, and high vascularization (McGuinness and Sapey, 2017). To counteract these risks, pulmonary defense mechanisms are activated, with the airway epithelium secreting protective substances such as mucins, lysozyme, nitric oxide, and cytokines to safeguard the respiratory tract against microbial invasion (Moldoveanu et al., 2009), though these responses can also trigger inflammatory processes.

Conditions such as asthma and chronic obstructive pulmonary disease (COPD) involve chronic inflammation but are characterized by distinct immune cell profiles: eosinophils predominate in asthma, whereas COPD involves neutrophils, macrophages, and T lymphocytes (Moldoveanu et al., 2009). Pulmonary fibrosis, often resulting from persistent inflammation, leads to airway remodeling, recurrent infections, and progressive lung dysfunction, involving the activation of neutrophils, eosinophils, fibroblasts, and macrophages (Kirkham and Barnes, 2013; Aghasafari et al., 2019).

COPD is a common, preventable, and treatable disease, characterized by persistent and progressive airway obstruction driven by a chronic inflammatory response (Caramori et al., 2013; 2016). According to the World Health Organization, COPD ranks as the third leading cause of morbidity and mortality worldwide in 2024 (World Health Organization, 2025). Although historically considered primarily a smoking-related illness, this view has evolved, as only 25% of chronic smokers develop COPD by age 80 (Caramori et al., 2016), and approximately 30% of patients have never smoked (Yoneyama et al., 2020). This highlights COPD as a multifactorial disease influenced by environmental exposures and genetic predispositions (Hogg et al., 2004; Margelidon-Cozzolino et al., 2016). Contributing factors in non-smokers include biomass smoke, air pollution, occupational dust, agricultural pesticide exposure, malnutrition, infections, airway hyperreactivity, and asthma (Mannino et al., 2006; Bagdonas et al., 2015).

The pathophysiology of COPD involves small airway obstruction, chronic inflammation, fibrosis, mucus hypersecretion, and emphysema, which collectively contribute to progressive airflow limitation through airway remodeling and destruction of lung parenchyma, reducing elasticity and increasing resistance (Caramori et al., 2016; Margelidon-Cozzolino et al., 2016). Oxidative stress plays a central role in COPD pathogenesis and occurs when endogenous antioxidant defenses fail to counteract the effects of reactive oxygen species (ROS). These ROS damage lung tissue by reacting with DNA and macromolecules, thereby promoting chronic pulmonary inflammation, a key aspect of COPD progression (Kirkham and Barnes, 2013; McGuinness and Sapey, 2017).

In COPD, inflammation is characterized by heightened recruitment and activation of immune cells, including macrophages, neutrophils, and lymphocytes, leading to increased pro-inflammatory mediators and reduced anti-inflammatory signals such as IL-10 (Caramori et al., 2013; 2016; Xu et al., 2024). Neutrophils secrete proteases that damage alveolar walls, while alveolar macrophages show increased activation and predominance of the pro-inflammatory M1 phenotype, resulting in greater ROS production and cytokine release.

Furthermore, COPD is often associated with intestinal microbial dysbiosis and impaired immune responses. Emerging evidence suggests that gut microbiota and their metabolites influence lung health, potentially contributing to COPD development and progression through systemic inflammatory mechanisms (Kirschner et al., 2021). Nutritional interventions have gained attention as potential strategies for COPD prevention, with antioxidant-rich diets shown to slow disease progression and reduce exacerbations (Tramontano and Palange, 2023).

Although *C. vulgaris* has shown antioxidant and anti-inflammatory activity, its toxicological safety and protective effects in a COPD model remain unexplored. Given this context, the present study aimed to characterize the bioactive compounds, phenolic profile, and *in vitro* antioxidant capacity of a hydro-methanolic extract of *C. vulgaris*. Moreover, we investigated its *in vivo* effects in two phases: first, by evaluating blood parameters, hepatic antioxidant enzymes, and cytochrome P450 activity in mice fed with *C. vulgaris* supplementation, and second, by assessing its potential preventive role in a murine model of chronic pulmonary inflammation, focusing on both local and systemic benefits.

## 2 Materials and methods

### 2.1 Preparation of the microalgal extract

Organic *C. vulgaris* powder (KoRo Handels GmbH, Berlin, Germany), whose nutritional values are reported in Table 1, was subjected to methanolic extraction. In detail, 2 g of the sample were weighed in triplicate and subjected to double extraction with 10 mL of 80% (v/v) methanol in continuous stirring for 2 h, in the dark. Later, the sample was centrifuged for 20 min at 3,000 rpm at room temperature using a Jouan CR3i centrifuge (Newport Pagnell, UK) and the supernatant was collected. The pellet was used for further extraction with additional 10 mL of 80% (v/v) methanol by stirring for 2-h in the dark. The supernatants of the two extractions were pooled (final concentration of 100 mg/mL) and stored at −20 °C until use (Qasim et al., 2016; Georgiopoulou et al., 2023).

**TABLE 1:** Chemical composition of *Chlorella vulgaris* powder.

Nutritional values	Per 100 g of product
Energy	327 kcal/1370 Kj
Fats	8.6 g
of which saturated fats	1.2 g
Carbohydrates	8.8 g
of which sugars	3.7 g
Fiber	3.2 g
Proteins	58 g
Salt	0 g

### 2.2 Antioxidant profiling of *Chlorella vulgaris*

#### 2.2.1 Determination of bioactive compounds

Total polyphenols content was quantified by the Folin–Ciocalteu colorimetric method (Singleton et al., 1999), measuring spectrophotometrically the absorbance at 760 nm and reported as mg of gallic acid equivalents per gram of dry weight (mg GAE/g dw). Aluminium chloride method was used to calculate total flavonoid content following the procedure by Kim et al. (2003) determining spectrophotometrically the absorbance at 430 nm and expressed as mg catechin equivalent per gram of dry weight (mg CE/g dw). The quantification of flavonols, expressed in terms of mg quercetin equivalent per gram of dry weight (mg QE/g dw) was carried out according to the procedure described by Romaní et al. (1996) recording spectrophotometrically the absorbance at 360 nm. The different pH spectrophotometric method described by Lee et al. (2005) was used to quantify total monomeric anthocyanins and expressed as mg cyanidin-3-glucoside per gram of dry weight (C3GE/g dw) measuring absorbance at 520 and 700 nm by spectrophotometer. Photosynthetic pigments, chlorophylls A and B and carotenoids were determined spectrophotometrically using the method of Lichtenthaler and Wellburn (1983), recording absorbance at 645 and 663 nm for chlorophylls and also at 460 nm for carotenoids, and expressed as mg per gram of dry weight (mg/g dw) following the formulas detailed in our previous work (Souid et al., 2024).

#### 2.2.2 Phenolic compounds profiling by UHPLC-ESI-MS/MS analysis

A selection of phenolic compounds were chosen for a thorough quantitative analysis of the extracts using a Sciex 5500 QTrap + mass spectrometer (AB Sciex LLC, Framingham, MA, USA) via Ultra-High-Performance Liquid Chromatography with Electrospray Ionization Tandem Mass Spectrometry (UHPLC-ESI-MS/MS), equipped with a Turbo V ion-spray source and coupled to an ExionLC AC System, custom-made by Shimadzu (Shimadzu Corporation, Kyoto, Japan) which includes two ExionLC AC pumps, autosampler, controller, degasser and tray. MS/MS experiments were performed in the electrospray negative ion mode with nitrogen serving as collision gas. The operational source parameters included source type as turbo spray, nebulizer gas (GS1) 70, turbo gas (GS2) 50, curtain gas (CUR) 10, temperature (TEM) 500 °C, Ionspray Voltage (IS) −4500 V, and entrance potential (EP) 10 V. Compounds parameters such as declustering potential (DP), collision energy (CE), and collision cell exit potential (CXP) were fine-tuned for the specific Selected Reaction Monitoring (SRM) transition on each component. Analyses were performed in triplicate and results expressed as µg per 100 g of dry weight (µg/100 g dw).

#### 2.2.3 *In vitro* antioxidant activity assays

The *in vitro* antioxidant activity of *C. vulgaris* extract was evaluated using a combination of fluorimetric and spectrophotometric methods. The determination of the oxygen radical absorbance capacity (ORAC) for the *C. vulgaris* extract followed the procedure outlined by Bacchiocca et al. (2006). AAPH was used as a peroxyl radical generator, with fluorescein as a probe. The DPPH radical scavenging activity was evaluated according to the procedure reported by Boudjou and co-workers (Boudjou et al., 2013). The total antioxidant power of *C. vulgaris* extract was determined using the ferric reducing antioxidant power (FRAP) assay, a colorimetric method based on the reduction of a ferric tripyridyltriazine complex to its ferrous form, as described by Benzie and Strain (1999). Trolox was used as the antioxidant standard, and the results were expressed in µg of Trolox equivalents (TE)/g dw.

### 2.3 Toxicological study

#### 2.3.1 Animal treatment

The *in vivo* experiment was conducted using thirty BALB/c male mice with a body weight range of 23–28 g. The animals were maintained in cages under a 12 h light/dark cycle at room temperature with relative humidity maintained at 55% and received food and drinking water *ad libitum*. The animals were divided into three groups of ten animals each and then treated for 4 weeks. The control group was fed a standard diet with 22.9% casein (CTR), a second group was fed a diet integrated with 1% of *C. vulgaris* (CV1%) and a third group was fed a diet integrated with 8% of *C. vulgaris* (CV8%). All diets were isocaloric and isoproteinogenic to the control diet (CTR) (20.2%, crude protein; 8%, crude fat; 7.2%, crude fiber; 4.8%, crude ash; 35.6%, starch; 6%, sugar) (ssniff Spezialdiäten GmbH, Soest, Germany). Animals body weight was monitored weekly alongside daily measurements of food consumption. At the end of experiment, blood was drawn by a cardiac puncture and centrifuged at 3,000 rpm for 10 min, then serum was stored at −20 °C until analyses. Then, the mice were euthanized through administration of a lethal overdose of 5% isoflurane. Liver tissue for gene expression analyses and microsomal preparation was removed and stored at −80 °C. All animal procedures of the present experiment [Authorization N. 575/2022-PR (protocol N.65E5B.66)] were performed with the approval of the Local Ethics Committee according to the Italian law regulating the use and human treatment of animals for the scientific purpose (legislative decree 26/2014), and the European Union Directive 2010/63/EU for animal experiments.

#### 2.3.2 Analysis of plasma biochemical parameters

The level of urea, creatinine, blood urea nitrogen (BUN), enzymatic activities of alanine aminotransferase (ALT) and aspartate aminotransferase (AST) were determined in accordance with the manufacturer’s instructions, measured utilizing a commercial assay made in specialized laboratory (PAIM Biolabor, Livorno, Italy).

#### 2.3.3 Microsome preparation and evaluation of hepatic enzyme activities

Microsomal and 10,000 rpm supernatant fractions were prepared as previously described (Longo et al., 1991). Protein content was determined according to the method described by Bradford (Bradford, 1976), using bovine serum albumin as standard. The effect of *C. vulgaris* on the xenobiotic metabolizing cytochrome P450 enzymes (CYP) was investigated analyzing the ethoxycoumarin-O-deethylase (ECOD), ethoxyresorufin-O-deethylase (EROD), aniline hydroxylase (AnH) and benzyloxyquinoline debenzylase (BQD) activities. ECOD and EROD activities were measured fluorimetrically by monitoring the formation of 7-hydroxycoumarin (Aitio, 1978) and resorufin (Lubet et al., 1985), respectively. The results were expressed as nmol of umbelliferone/mg protein x min and pmol/mg protein x min, respectively. The determination of AnH was carried out using the protocol reported by Ko et al. (1987) by detecting the production of p-aminophenol by CYP2E1 and, to a lesser extent, by CYP1A2. The results were expressed as pmol/mg protein x min. The determination of BQD activity by CYP3A following the debenzylation reaction of benzyloxy quinoline to 7-hydroxyquinoline was carried out using the protocol reported by Stresser et al. (2000). The results were expressed as pmol/mg protein x min. The activity of catalase (CAT) was determined using the protocol reported by Aebi (1984) and its degradation was detected by following the decrease in absorbance at 240 nm for 1 min. The enzyme activity was expressed as nmol H_2_O_2_/mg of protein x min. The determination of glutathione reductase (GSR) activity was conducted using the method by Wheeler et al. (1990). The activity of glutathione reductase was monitored by a spectrophotometer at 340 nm by evaluating the consumption of NADPH. The results were expressed as nmol/mg protein x min. The DT-diaphorase activity was investigated by measuring the reduction in dichlorophenol-indophenol at 630 nm, following the method described by Benson et al. (1980). The results were reported as nmol/mg protein x min.

### 2.4 Protective assessment in a COPD mouse model

#### 2.4.1 Animal treatment

The *in vivo* experiment was conducted using forty BALB/c male mice with a body weight range of 23–28 g. The animals were maintained in cages under a 12 h light/dark cycle at room temperature with relative humidity maintained at 55% and received food and drinking water *ad libitum*. The animals were divided into four groups of ten animals each and then treated for 4 weeks. The control group was fed a standard diet with 22.9% casein (CTR), the second group was induced with chronic obstructive pulmonary disease (COPD) and other two groups were induced with chronic obstructive pulmonary disease and also fed with a diet integrated with 1% or 8% of *C. vulgaris* (COPD + CV1% and COPD + CV8%, respectively) (see paragraph 2.3.1). COPD was induced in the animals via intranasal administration of porcine pancreatic elastase (PPE) (1.2 units on the first day of each week for the first 2 weeks) and lipopolysaccharides (LPS) (7 µg on the fourth day of each week for 4 weeks and on the first day of each week for the last 2 weeks) following the protocol of Kim and colleagues (Kim et al., 2017), slightly modified as specified above. The control group was treated for 4 weeks with phosphate-buffered saline (PBS), an aqueous saline solution containing NaCl (137 mM), KCl (2.7 mM), Na_2_HPO_4_ (10 mM), KH_2_PO_4_ (1.8 mM). To confirm successful induction of the COPD model, animals were monitored for clinical signs (body weight, respiratory distress, and reduced mobility), while at sacrifice, lung tissues were examined histologically by H&E staining; validation criteria included alveolar enlargement, wall thinning, peribronchial and perivascular inflammatory infiltrates, and mucus accumulation, consistent with previously described COPD phenotypes (Lee et al., 2025). Animals body weight was monitored weekly alongside daily measurements of food consumption. At the end of experiment, mice were anesthetized by 3% isoflurane and underwent bronchoalveolar lavage fluid (BALF) collection through tracheal cannula using sterile PBS. The fluid was then frozen in liquid nitrogen and stored at −80 °C until it was analyzed for neutrophils, eosinophils, monocytes-macrophages, lymphocytes, plasma cells, epithelial cells, mucus, red blood cells (RBC) and total inflammatory cells by a specialized laboratory (PAIM Biolabor, Livorno, Italy). The animals were subsequently euthanized by administering a lethal dose of 5% isoflurane. Then, caecal content was collected from each animal and stored at −80 °C and lungs were partly preserved in a 70% mixture of ethyl and isopropyl alcohol (approximately 60% and 40%, respectively) and 30% distilled water solution at 4 °C for histopathological analysis and partly stored at −80 °C for gene expression studies. All animal procedures of the present experiment (Authorization N. 575/2022-PR (protocol N.65E5B.66) were performed with the approval of the Local Ethics Committee according to the Italian law regulating the use and human treatment of animals for the scientific purpose (legislative decree 26/2014), and the European Union Directive 2010/63/EU for animal experiments.

#### 2.4.2 BALF analysis

The Broncho-Alveolar Lavage Fluid (BALF) analyses allowed the scoring of total inflammatory cells involved in the onset and progression of the disease, in particular neutrophil granulocytes, monocytes-macrophages, lymphocytes, and plasma cells. Other key parameters such as mucus, RBC, and epithelial cells were also evaluated. For each parameter, a cell count was performed, and a score was assigned on a scale from zero (absence) to three (high content). The final “score” was calculated as the sum of all values recorded for each sample. The analyses were conducted by a specialized laboratory (Paim Biolabor, Livorno, Italy).

#### 2.4.3 Histopathological analysis

The lungs were collected and preserved in a 70% alcohol solution as described above. The samples were subjected to a progressive dehydration process using increasing concentrations of ethanol solutions, followed by xylene, as follows: 70% ethanol overnight, 75% ethanol for 30 min, 95% ethanol for 75 min, 100% ethanol for 60 min (repeated twice), and xylene for 45 min twice. Trimming was performed according to laboratory standard operating procedures (SOPs). Subsequently, lung samples were processed and embedded in paraffin blocks using the Bec150 inclusion unit (Bio-Optica, Milan, Italy) in accordance with SOPs. Tissue sections, 5 µm thick, were cut using a Supercut 2050 microtome (Reichert-Jung, NY, USA) and stained with haematoxylin and eosin. Finally, all sections were examined under a microscope. In addition, for each group, two images were considered, and the area of the air spaces was measured in all visible alveoli, with the average being calculated.

#### 2.4.4 Biomarkers of oxidative stress in the lungs

The concentration of malondialdehyde (MDA) and glutathione (GSH) levels in lung samples were quantified following the protocols described by Browne and Armstrong (1998), Seljeskog et al. (2006), respectively, with slight modifications. Particularly, concerning MDA, an aliquot of 100 µL homogenate was mixed with 0.1125 N PCA (300 µL) and 40 mM TBA (300 µL), incubated in a boiling water bath for 60 min, then cooled at −20 °C for 20 min. Methanol (600 µL) and 20% TCA (200 µL) were added, and the suspension was centrifuged (7000× g, 6 min). MDA content in the supernatant was quantified fluorometrically (Perkin Elmer LS-45, λex = 525 nm, λem = 560 nm) using a standard curve of hydrolyzed TEP (0.52–33.5 µM). Results are expressed as nmol MDA/g tissue. Regarding GSH, in detail, proteins were precipitated with 10% TCA at 4 °C for 30 min. Aliquots (150 µL) were incubated with o-phthaldehyde (1 mg/mL in 10% methanol, v/v) for 15 min at 37 °C and centrifuged (625× g, 3 min). Fluorescence was measured (Perkin Elmer LS-45, λex = 350 nm, λem = 420 nm) using a GSH standard curve (0.78–50 µM). Results are expressed as μmol GSH/g tissue.

### 2.5 Gene expression analysis

The total RNA was isolated from approximately 30 mg of frozen mouse liver or lung tissue using the Aurum™ Total RNA Mini Kit (Bio-Rad Laboratories, Inc., California, United States) according to the manufacturer’s protocol. Isolated RNA samples were stored at −80 °C until further analysis. Reverse transcription of total RNA was conducted using the iScript™ gDNA Clear cDNA synthesis Kit (Bio-Rad Laboratories, Inc., California, United States), and the resulting cDNA samples were stored at −20 °C. Quantitative real-time PCR (qRT-PCR) was performed with the SsoAdvanced™ Universal SYBR^®^ Green Supermix (Bio-Rad, California, USA) on a CFX Connect Real-Time PCR Detection System (Bio-Rad, California, United States). The amplification program recommended in the manufacturer’s manual was employed. Primer pairs for CYP2C29, CYP2E1, CYP3A25 for liver, TGFβ for lungs and the endogenous reference genes β-actin, GAPDH and B2M for both liver and lungs were designed using Primer3Plus software and synthesized by Metabion International AG (Planegg, Germany). The expression stability of the housekeeping genes was assessed in liver tissues using RT-qPCR. Ct values remained consistent across all samples and conditions, with minimal variability, and their expression was unaffected by treatments, confirming their suitability as reliable internal controls. Reference sequences for all mouse genes were obtained from GenBank, and reference genes served as the endogenous control to normalize RNA input and potential variability in reverse transcription efficiency. Relative gene expression levels were calculated by normalizing target gene expression to the mean expression of the control group (assigned a value of 1). All genes were analyzed in triplicate, and relative expression levels were determined using the 2^−ΔΔCT method. Results were expressed as fold changes in expression relative to control samples. More details about these primers are present in Table 2.

**TABLE 2:** Primer pairs, sequence, and melting temperature for real time PCR experiments.

Target gene	Direction	Sequence (5′-3′)	Temperature
CYP2C29	Forward	ATT CAT CGA CCT CCT CCC CA	60 °C
	Reverse	AAA GTG CCC AGG GTC AAA CA	58 °C
CYP2E1	Forward	TCA CTG GAC ATC AAC TGC CC	60 °C
	Reverse	TGT GCT GGT GGT CTC TGT TC	60 °C
CYP3A25	Forward	GGG TTT TAT GAG GGC CCA CA	60 °C
	Reverse	TTC ATG AAT CCC ACT GGC CC	60 °C
TGFβ	Forward	ACT GGA GTT GTA CGG CAG TG	63 °C
	Reverse	GGG GCT GAT CCC GTT GAT TT	63 °C
β-actin	Forward	TAC TGC TCT GGC TCC TAG CA	60 °C
	Reverse	CGG ACT CAT CGT ACT CCT GC	63 °C
GAPDH	Forward	TGA TGG GTG TGA ACC ACG AG	60 °C
	Reverse	AGT GAT GGC ATG GAC TGT GG	60 °C
B2M	Forward	GGT GAC CCT GGT CTT TCT GG	63 °C
	Reverse	TGT TCG GCT TCC CAT TCT CC	60 °C

### 2.6 Statistical analysis

All the results are expressed as the mean value (n = 3) ± standard deviation (SD). In the *in vivo* studies, significant differences among means of the groups were highlighted through the one-way analysis of variance (ANOVA) followed by Tukey’s test, with p ≤ 0.05. Statistical analyses were performed using Prism, GraphPad Software (San Diego, CA, United States). After variables transformation, a Pearson’s correlation analysis was performed to assess the relationships between the four experimental groups (CTR, COPD, COPD + CV1% and COPD + CV8%) and various variables, including BALF cells, caecal content and oxidative stress markers. Moreover, a principal component analysis (PCA) was performed to process the data from each animal in the *in vivo* study, in particular BALF cells, SCFAs of caecal content, and markers of oxidative stress status in the lungs. Pearson’s correlation analysis and PCA were performed using XLSTAT software (Version 2019).

## 3 Results

### 3.1 Bioactive compounds and antioxidant capacity of *Chlorella vulgaris*

The methanolic extract of *C*. *vulgaris* exhibited a total phenolic content of 1.08 ± 0.01 mg GAE/g dry weight (dw), along with 9.06 ± 0.65 mg CE/g dw of flavonoids, 30.91 ± 2.26 mg QE/g dw of flavonols, and 0.10 ± 0.02 mg C3GE/100 g dw of anthocyanins (Table 3). The concentrations of chlorophyll a and chlorophyll b were 1.84 ± 0.03 mg/g dw and 1.25 ± 0.02 mg/g dw, respectively, while the carotenoid content reached 225.86 ± 2.37 mg/g dw, representing the most abundant group of compounds in the extract (Table 3).

**TABLE 3:** Bioactive compounds and *in vitro* antioxidant activity of *Chlorella vulgaris.*

		C. *vulgaris*
Bioactive compounds	Total polyphenols (mg GAE/g dw)	1.08 ± 0.01
	Flavonoids (mg CE/g dw)	7.43 ± 1.31
	Flavonols (mg QE/g dw)	30.91 ± 2.26
	Anthocyanins (mg C3GE/g dw)	0.10 ± 0.02
Pigments	Chlorophyll a (mg/g dw)	1.84 ± 0.03
	Chlorophyll b (mg/g dw)	1.25 ± 0.02
	Carotenoids (mg/g dw)	225.86 ± 2.37
Antioxidant activity	ORAC (µg TE/g dw)	27.17 ± 1.73
	DPPH (µg TE/g dw)	2.39 ± 0.10
	FRAP (µg TE/g dw)	652.77 ± 22.27

Values are reported as means ± standard deviation (SD) of three replicates (n = 3).

To further assess the antioxidant potential of the methanolic extract, three *in vitro* assays were conducted. The ORAC assay yielded 27.17 ± 1.73 μmol TE/g dw. The DPPH radical scavenging assay revealed an antioxidant activity of 2.39 ± 0.10 μg TE/g dw. The FRAP assay indicated a strong reducing capacity, with a value of 652.77 ± 22.27 mg TE/g dw.

### 3.2 Quantification of phenolic compounds in *Chlorella vulgaris* extract by UHPLC-ESI-MS/MS

The phenolic composition of *C. vulgaris* methanolic extract was characterized using UHPLC-ESI-MS/MS, revealing a total of 37 phenolic compounds (Table 4). Flavonols were the most abundant class, accounting for 293.41 μg/100 g dry weight (dw), with quercetin being the predominant compound (241.16 μg/100 g dw). Eight phenolic acids were identified, the most abundant being gallic acid (53.58 μg/100 g dw), vanillic acid (39.83 μg/100 g dw), and protocatechuic acid (19.98 μg/100 g dw). The extract also contained notable quantities of flavan-3-ols (110.97 μg/100 g dw), with catechin and epicatechin being the major representatives. Additionally, procyanidins (63.07 μg/100 g dw), oleuropein (51.02 μg/100 g dw), and verbascoside (13.80 μg/100 g dw) were identified. Minor compounds included stilbenoids (6.97 μg/100 g dw) and anthocyanins (6.70 μg/100 g dw).

**TABLE 4:** Content of individual phenolic compounds in the hydro-methanolic extract of *Chlorella vulgaris.*

Phenolic compound	Acronym	*C. vulgaris* (µg/100 g dw)
Gallic acid	GA	53.58 ± 2.03
Protocatechuic acid	PRA	19.98 ± 1.98
3-O-Caffeoylquinic acid (Chlorogenic acid)	3CQA	0.94 ± 0.07
Caffeic acid	CA	3.15 ± 0.22
Vanillic acid	VA	39.83 ± 3.01
*p*-Coumaric acid	*p*CA	0.68 ± 0.10
*trans*-Ferulic acid	*t*FA	0.09 ± 0.00
2,3-Dicaffeoyl-tartaric acid (Chicoric acid)	DCT	2.50 ± 0.33
**∑ Phenolic acids** ^*^		**120.75**
Quercetin	Q	241.16 ± 10.33
Quercetin 3-O-glucoside	Q3G	6.15 ± 1.02
Quercetin 3-O-rutinoside (Rutin)	Q3R	24.74 ± 3.42
Quercetin 3,4-O-diglucoside	QDG	0.22 ± 0.01
Quercetagetin 7-O-glucoside	QA7G	4.05 ± 0.82
Kaempferol 7-O-glucoside	K7G	5.15 ± 1.02
Kaempferol 3-O-glucoside	K3G	10.67 ± 1.99
Kaempferol 3-O-rutinoside	K3R	1.27 ± 1.98
**∑ Flavonols**		**293.41**
Cyanidin 3-O-glucoside (Kuromanin)	C3G	2.23 ± 0.82
Cyanidin 3,5-O-diglucoside (Cyanin)	CDG	0.30 ± 0.04
Peonidin 3,5-O-diglucoside	PDG	N.d
Malvidin 3-O-glucoside (Oenin)	M3G	0.46 ± 0.05
Petunidin 3-O-glucoside	Pt3G	0.67 ± 0.09
**∑ Anthocyanins** ^*^		**6.70**
(+) -Catechin	C	56.06 ± 4.91
(−) -Epicatechin	EC	54.91 ± 6.29
**∑ Flavan-3-ols** ^*^		**110.97**
Procyanidin B1	PCB1	3.33 ± 1.02
Procyanidin B2	PCB2	32.92 ± 2.91
Procyanidin B3	PCB3	26.82 ± 3.01
**∑ Procyanidins** ^*^		**63.07**
Resveratrol 3-O-glucoside (Piceid)	R3G	0.35 ± 0.03
Resveratrol	RES	2.30 ± 0.87
**∑ Stilbenoids** ^*^		**6.97**
Oleuropein	OLE	51.02 ± 3.22
Verbascoside	VER	13.80 ± 2.25
Ligstroside	LIG	4.57 ± 1.88
Hydroxytyrosol	HYT	2.41 ± 0.72
Eriodictyol	ERI	1.11 ± 0.63
Naringenin	NEN	0.27 ± 0.04
Phloridzin	PHZ	0.23 ± 0.03
Phloretin	PHL	0.11 ± 0.01
**∑ Others** ^*^		**74.14**

Values are reported as means ± SD, of three replicates (n = 3). N.d.: not detected. * Sum of phenolic acids, flavonols, anthocyanins, flavan-3-ols, procyanidins, stilbenoids and other compounds determined using HPLC-MS are shown in bold.

### 3.3 *In vivo* toxicological assessment of *Chlorella vulgaris*

#### 3.3.1 Effect of treatments with *Chlorella vulgaris* on the animal body and food intake

**FIGURE 1:**
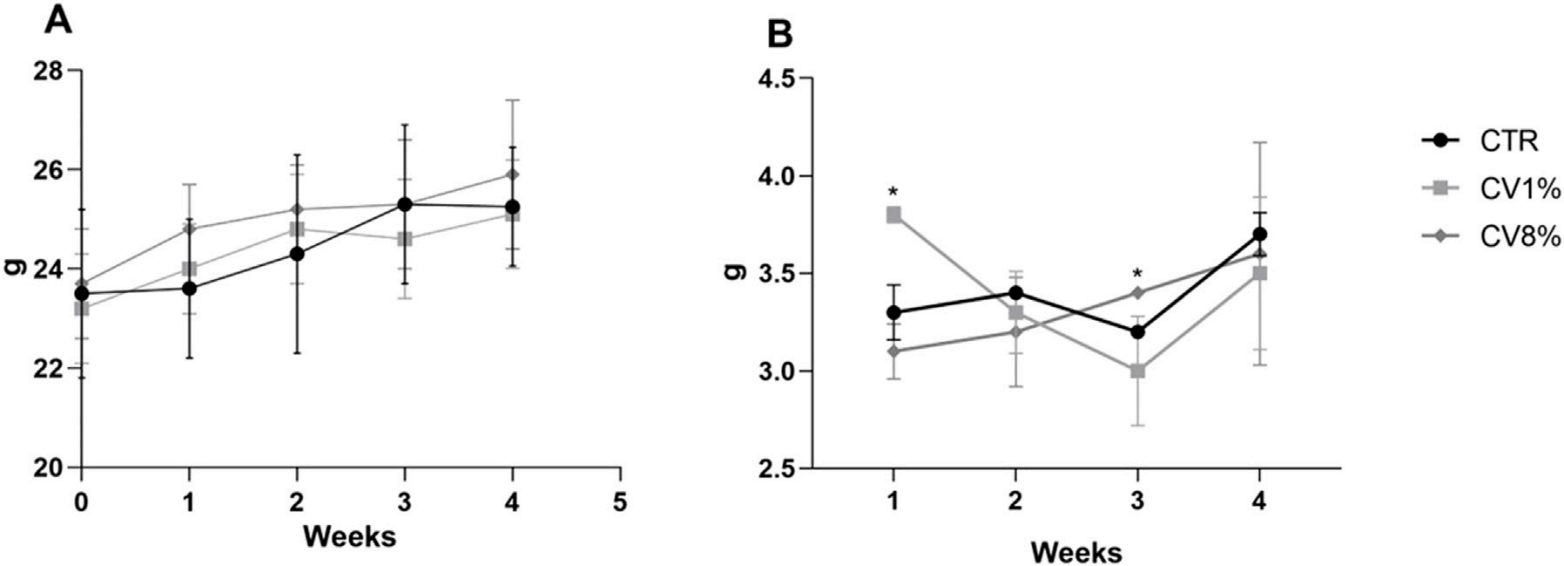
Animals body weights **(A)** and daily food consumption **(B)** for C. vulgaris dietary supplementation toxicological study of the CTR (•), CV1% (◇) and CV8% (◇) groups. Values are expressed means ± SD.

During the experiment for the toxicological study of *C. vulgaris* dietary supplementation the animals were weighed weekly. The average body weight of each group over time is shown in Figure 1A. In all groups, the weight of mice slightly increased over the weeks and no significant differences were noted among groups. The daily food consumption monitored over the weeks is shown in Figure 1B. Although they do not seem to have any noteworthy significance, statistically significant differences in daily food consumption among the groups of animals during the first and third week of treatment were noted in the dietary supplementation toxicological study, probably due to the different palatability of the diets.

#### 3.3.2 Plasma biochemical parameters

To assess the safety profile of *C. vulgaris*, liver and kidney toxicity markers were evaluated in the plasma of mice (Table 5). The groups treated with both 1% and 8% *C. vulgaris* diets showed a reduction in plasma levels of aspartate aminotransferase (AST), alanine aminotransferase (ALT), and creatinine, compared to the control group, indicating no evidence of hepatotoxicity or nephrotoxicity at the tested concentrations and, indeed, a potential protective effect. A slight increase in urea and blood urea nitrogen (BUN) levels was observed in *C. vulgaris*-treated animals relative to the control group, with the effect being more pronounced in the 1% group than in the 8% group. However, this increase was not statistically significant. These findings support the absence of systemic toxicity associated with dietary supplementation of *C. vulgaris* at both tested concentrations.

**TABLE 5:** Biochemical parameters in the mice plasma of CTR, CV1% and CV8% groups.

	CTR (n = 10)	CV1% (n = 10)	CV8% (n = 10)
AST (U/l)	284^a^ ± 87.9	189.4^a^ ± 76.2	203.8^a^ ± 68.7
ALT (U/l)	33^a^ ± 5.7	20.4^b^ ± 3.5	22.8^b^ ± 4.1
Urea (mg/dL)	39.5^a^ ± 9.4	57.4^a^ ± 16.6	45.7^a^ ± 8.5
BUN (mg/dL)	18.5^a^ ± 4.4	26.8^a^ ± 7.6	21.3^a^ ± 4.0
Creatinine (mg/dL)	0.22^a^ ± 0.09	0.13^b^ ± 0.02	0.12^b^ ± 0.01

Results are reported as means ± SD, of three replicates (n = 3). Values within each row different letters (a,b) are significantly different by one-way ANOVA, followed by Tukey’s post-test (p ≤ 0.05).

#### 3.3.3 Activity and expression levels of xenobiotic metabolizing system

**FIGURE 2:**
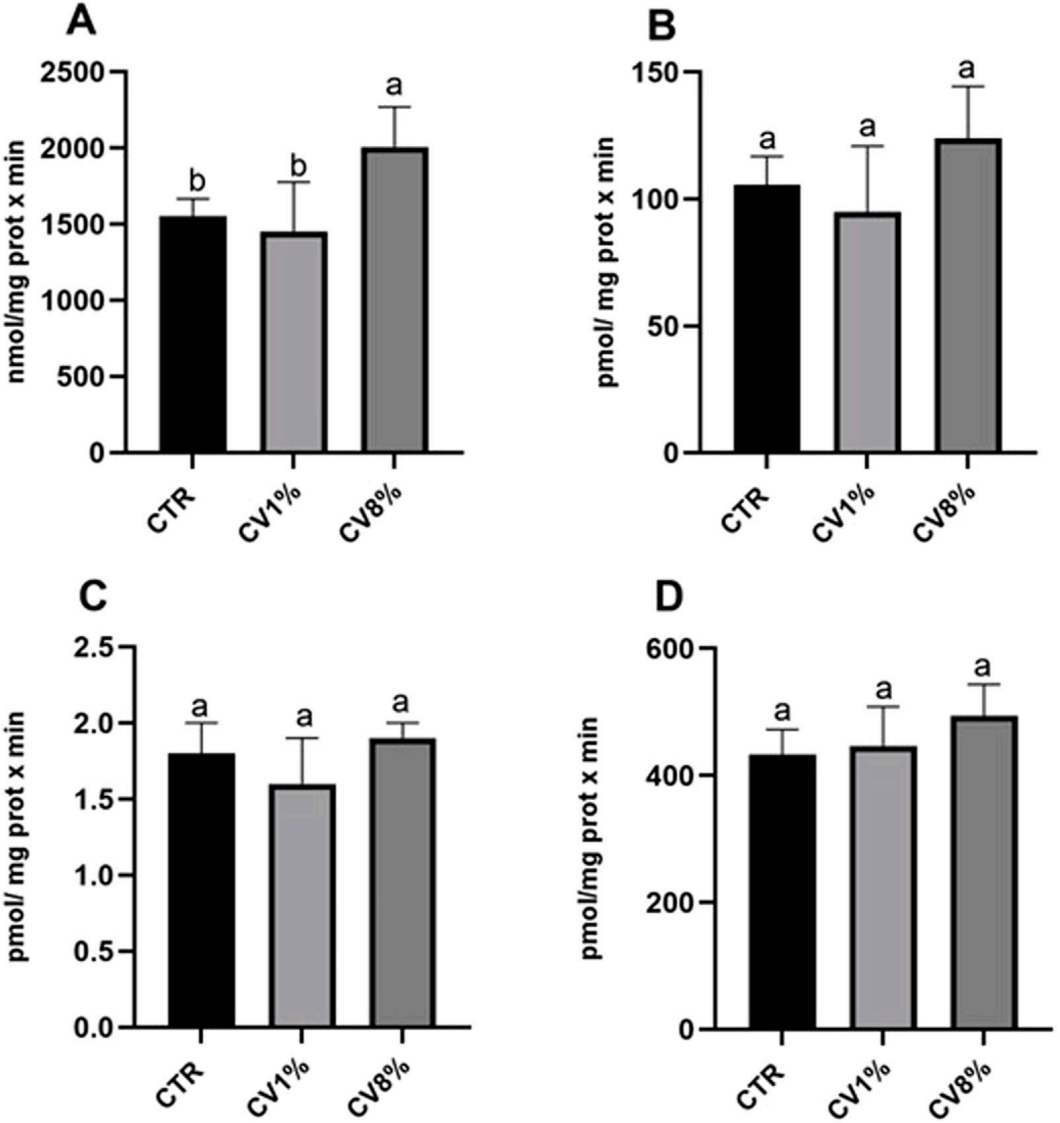
Ethoxycoumarin-O-deethylase (ECOD) **(A)**, ethoxresorufin-O-deethylase (EROD) **(B)**, aniline hydroxylase (AnH) **(C)** and benzyloxyquinoline debenzylase (BqD) **(D)** activities in liver of all CTR, 1% *C*. vulgaris and 8% *C*. vulgaris mice. CV: *C*. vulgaris. Values are expressed as means (n=3) ± SD and reported as nmol umbelliferone/ mg protein x min for ECOD, as pmol resorufin/ mg protein x min for EROD, as pmol p-aminophenol/mg prot x min for AnH and as pmol 7-hydroxyquinoline/mg prot x min for BZQ activity. a, b: values significantly different by one way ANOVA-test, followed by Barlett’s post-test (p ≤ 0.05).

**FIGURE 3:**
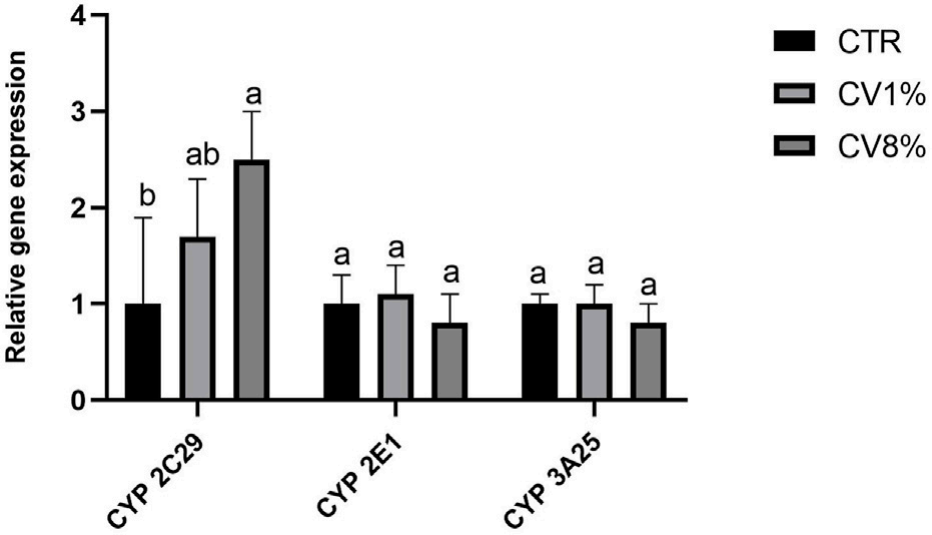
Relative gene expression of CYP2C29, CYP2E1 and CYP3A25 measured by real-time RT-PCR in liver from CTR, 1% C. vulgaris and 8% *C*. vulgaris mice. Data represent the means ± SD of mice from each group and are reported as relative gene expression compared to the control. Results are normalized for the expression levels of housekeeping gene β-actin, GAPDH and B2M and referred to the mean of the controls, to which a value of 1 was assigned. For each gene, the presence of different letters (a,b) indicates statistically significant differences among groups according to one-way ANOVA test, followed by Barlett’s post-test (p ≤ 0.05).

Figure 2 illustrates the effects of *C. vulgaris* dietary supplementation on the activity of enzymes involved in the xenobiotic metabolizing system. Specifically, the following enzymatic activities were assessed: ECOD, representing multiple cytochrome P450 isoforms; EROD, a marker of CYP1A1/1A2; AnH, indicative of CYP2E1; and BqD, associated with the CYP3A family (Vornoli et al., 2014). Mice fed an 8% *C. vulgaris*-supplemented diet showed a statistically significant increase in ECOD activity compared to those receiving the control diet or the 1% *C. vulgaris* diet. Slight, non-significant increases in EROD, BqD, and AnH activities were also observed in the 8% group. The 1% *C. vulgaris* group did not exhibit statistically significant changes in any of the assessed enzyme activities, with a slight, non-significant decrease observed in ECOD, EROD, and AnH, and a slight, non-significant increase in BqD, relative to the control. To further explore the regulation of xenobiotic metabolism, gene expression analysis of CYP2C29, CYP2E1, and CYP3A25 was performed by real-time PCR (Figure 3). A dose-dependent upregulation of CYP2C29 transcription was observed in both 1% and 8% *C. vulgaris*-treated groups, with a 2.5-fold increase in the higher dose group relative to the control. No significant changes were detected in the expression of CYP2E1 or CYP3A25.

#### 3.3.4 Activity and expression levels of antioxidant and detoxifying enzymes

**FIGURE 4:**
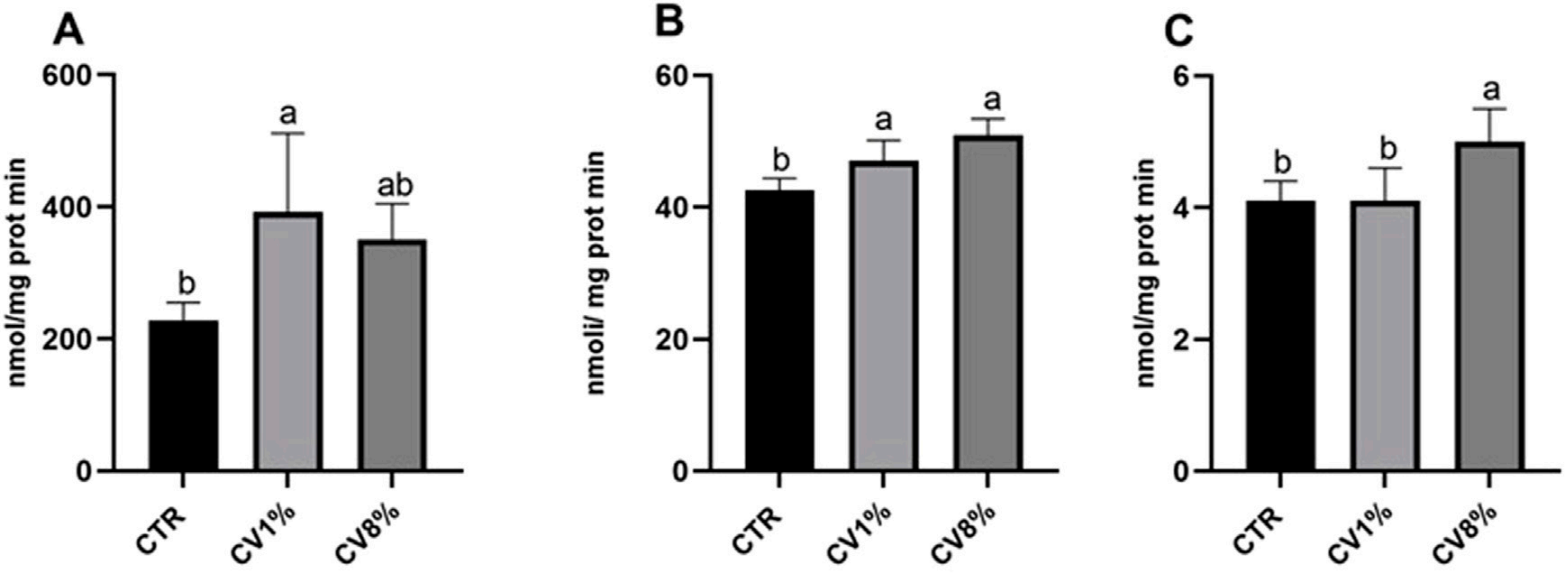
Catalase **(A)**, glutathione reductase **(B)** and DT-diaphorase **(C)** activities in the liver of all CTR, 1% *C*. vulgaris and 8% *C*. vulgaris mice. CV: *C*. vulgaris. Values are expressed as means (n=3) ± SD and reported as nmol/mg protein x min for catalase, as nmol/ mg protein x min for glutathione reductase and as nmol/mg prot x min for DT-diaphorase activity. a, b: values significantly different by one-way ANOVA test, followed by Barlett’s post-test (p ≤ 0.05).

Both 1% and 8% *C. vulgaris* dietary supplementation increased the enzymatic activity of CAT and GSR compared to the control group. The increase in CAT activity was statistically significant in the 1% *C. vulgaris* group, while GSR activity was significantly elevated at both supplementation levels. A statistically significant increase in DT-diaphorase activity was observed only in the 8% *C. vulgaris* group (Figure 4).

**FIGURE 5:**
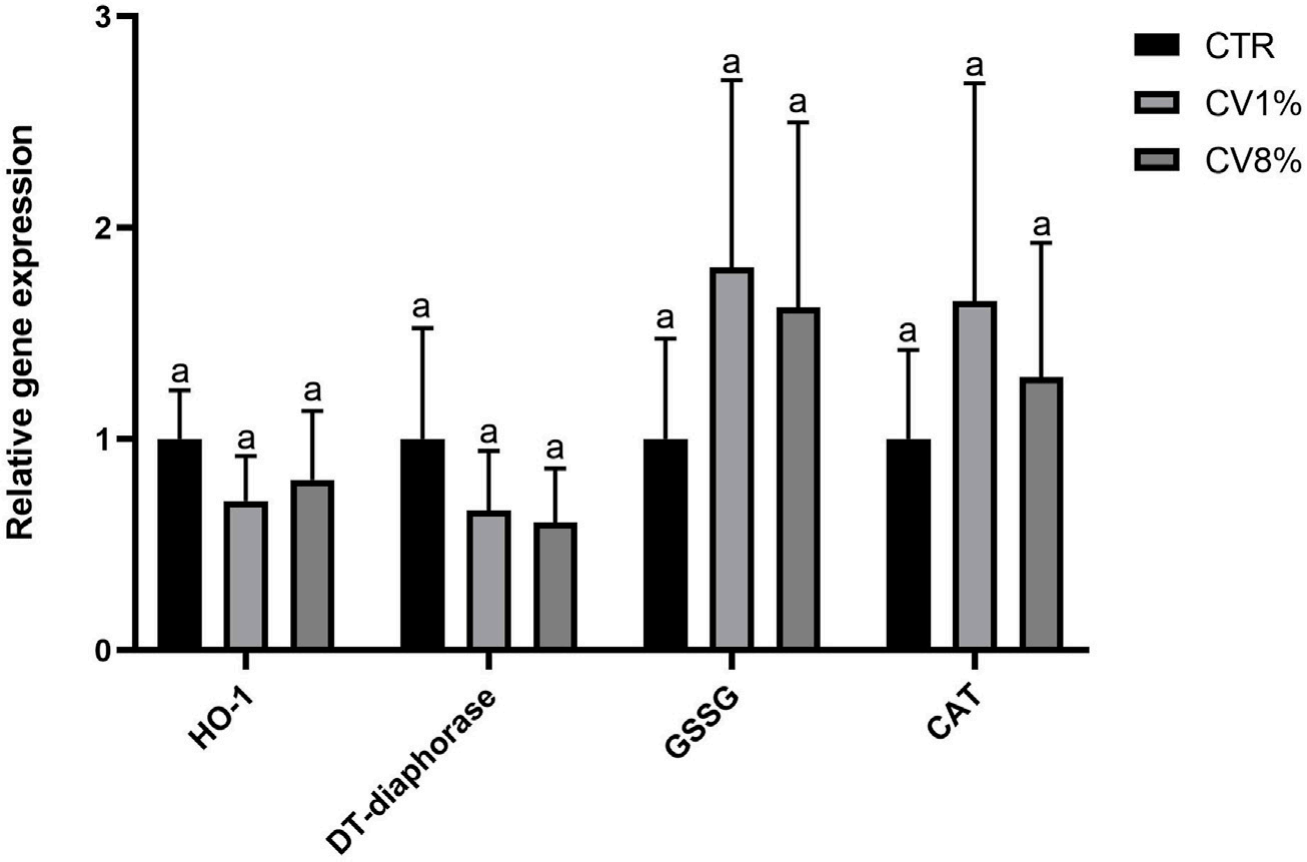
Relative genes expression of HO-1, DT-diaphorase, GSSG and CAT measured by real-time RT-PCR in liver from all CTR, 1% *C*. vulgaris and 8% *C*. vulgaris mice. Data represent the means ± SD of mice from each group and are reported as relative gene expression compared to the control. Results are normalized for the levels of housekeeping gene β-actin, B2M, GAPDH and referred to the mean of the controls, to which a value of 1was assigned. For each gene the presence of different letters (a,b) indicates significant differences among groups according to one-way ANOVA test, followed by Barlett’s post-test (p ≤ 0.05).

Gene expression analysis of antioxidant and detoxification enzymes (Figure 5) did not reveal statistically significant differences among the groups, likely due only to sample size (n = 10). Nonetheless, the upregulation trends in CAT and GSR gene expression observed in both *C. vulgaris*-fed groups were consistent with the corresponding increases in enzyme activity, reinforcing the biochemical findings.

### 3.4 *In vivo* evaluation of the potential preventive effect of *Chlorella vulgaris* in a COPD murine model

#### 3.4.1 Effect of treatments on the animal body and food intake

**FIGURE 6:**
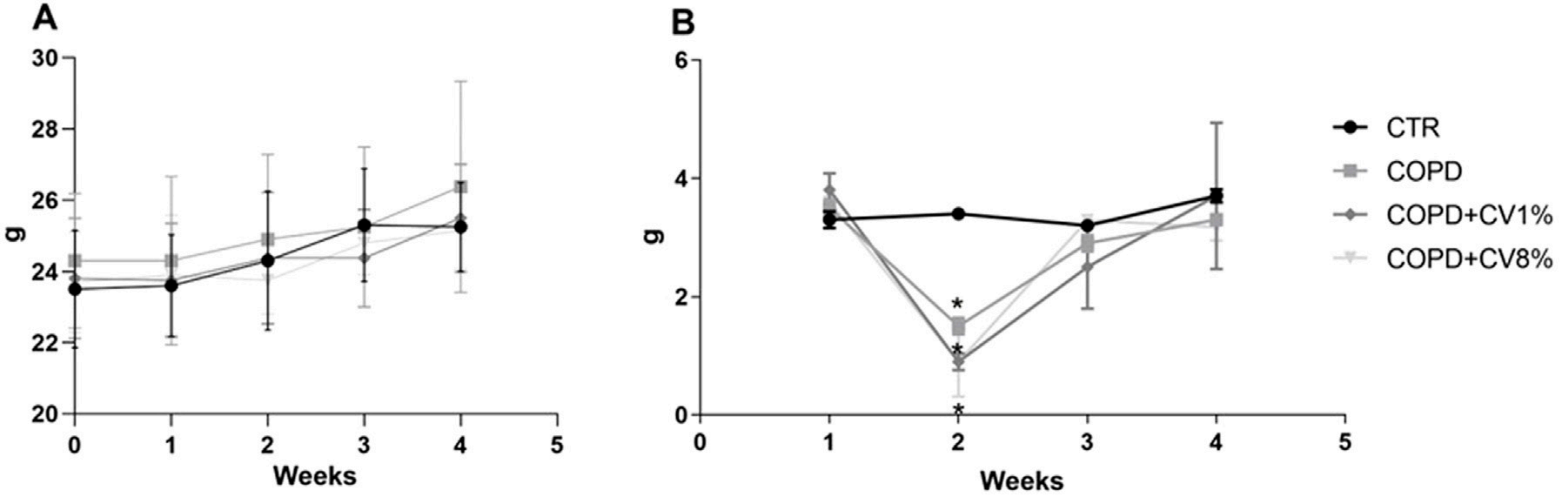
Animals body weights **(A)** and daily food consumption **(B)** of the study on *C*. vulgaris potential prevention against COPD of the CTR (•), COPD (▪), COPD+CV1% (◇), and COPD+CV8% (▼) groups. Values are expressed means ± SD.

The average body weight of each group over time is shown in Figure 6A for the study on *C. vulgaris* potential prevention on COPD. In all groups, the weight of mice slightly increased over the weeks and no significant differences were noted among groups. The daily food consumption monitored over the weeks is shown in Figure 6B. The animals with COPD, both in the presence and absence of *C. vulgaris* supplementation, showed significant variations in food consumption, likely due to COPD induction treatments. Specifically, a notable reduction in food intake was observed during the second week in comparison to the control group, followed by a subsequent increase in the following weeks, with intake levels returning to values approximating those of the control group.

#### 3.4.2 Modulation of BALF score

**FIGURE 7:**
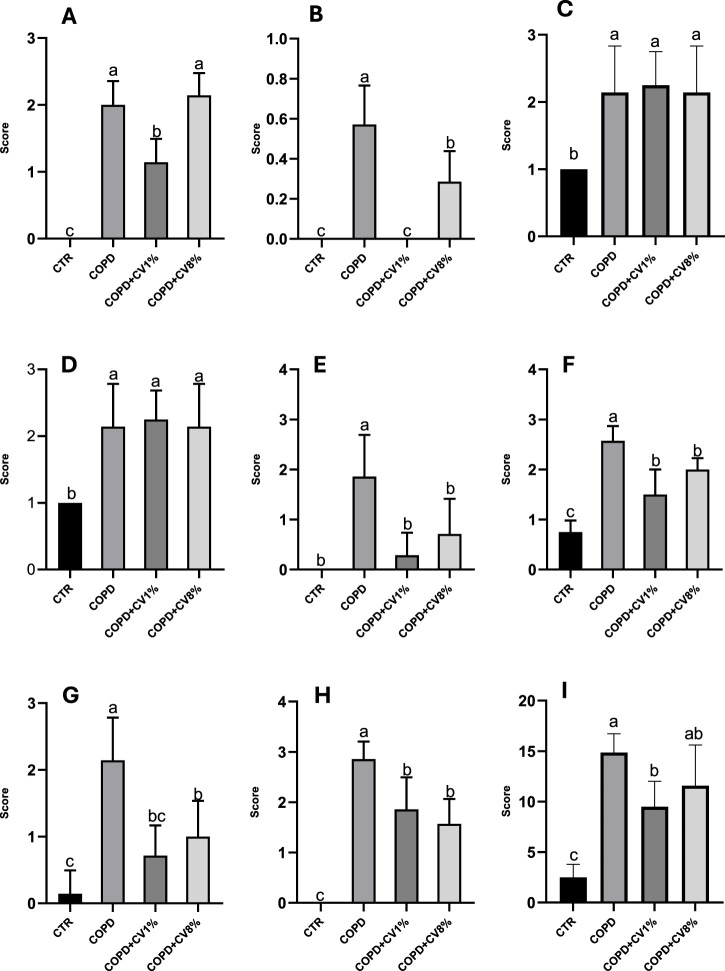
Score functional to cell count attributed to neutrophils **(A)**, eosinophils **(B)**, monocytes-macrophages **(C)**, lymphocytes **(D)**, plasma cells **(E)**, epithelial cells **(F)**, mucus **(G)**, red blood cells **(H)** and total inflammatory cells **(I)**, as the sum of all values recorded for each sample, in the BALF of all CTR, COPD, COPD+1% C. vulgaris and COPD+ 8% C. vulgaris. CV: C. vulgaris. The values are expressed as means ± SD. Different letters (a, b, c) indicate significant differences between the groups of mice according to one-way. A B C D E F G H I ANOVA test. (p ≤ 0.05). Then the Barlett’s post-test was carried out, except for subfigures A, B, E and H because some columns have values equal to zero.

To investigate the inflammatory profile associated with COPD, BALF was analyzed, and cell-type-specific scores were evaluated across experimental groups (Figure 7). Neutrophil infiltration, a recognized marker of airway inflammation, was significantly elevated in the COPD group compared to controls. Treatment with 1% *C. vulgaris* significantly reduced neutrophil counts relative to the COPD group, though levels remained higher than in controls. In contrast, 8% *C. vulgaris* did not reduce neutrophil infiltration, and levels remained significantly higher than in the control group (Figure 7A). Eosinophil scores were highest in the COPD group and significantly reduced by the 8% *C. vulgaris* treatment. No eosinophils were detected in either the control or the 1% *C. vulgaris* groups (Figure 7B). Macrophage scores were significantly elevated in all experimental groups relative to controls, with no significant differences observed among the 1%, 8%, and COPD groups (Figure 7C). A similar trend was observed for lymphocytes, with the control group showing significantly lower scores compared to all other groups (Figure 7D). The plasma cell score (Figure 7E) was significantly elevated in the COPD group compared to the control. Both microalgae treatments reduced plasma cell infiltration to levels statistically comparable with controls. Epithelial cell scores followed a similar trend (Figure 7F), with the COPD group exhibiting the highest values, which were significantly reduced in the microalgae-treated groups.

Mucus production, as assessed by scoring (Figure 7G), was significantly increased in the COPD group relative to controls. Both concentrations of *C. vulgaris* led to a statistically significant reduction in mucus, although only the 1% treatment restored levels comparable to controls. Red blood cell (RBC) infiltration in BALF (Figure 7H) was also elevated in the COPD group compared to the control. Treatment with both 1% and 8% *C. vulgaris* significantly reduced RBC presence. An overall inflammatory score, derived from the sum of all individual cell-type scores (Figure 7I), showed that the COPD group had significantly higher total inflammatory cell counts compared to controls. Both *C. vulgaris* treatments resulted in a reduction of this total score, with statistical significance achieved only for the 1% supplementation group.

#### 3.4.3 Evaluation of the protective effect of *Chlorella vulgaris* against COPD through histopathological analysis of the lungs

To evaluate the protective effects of dietary *C. vulgaris* supplementation on lung morphology in a COPD model, histological analysis was performed using hematoxylin-eosin staining, followed by quantitative assessment of alveolar size. In COPD, enlargement of alveolar spaces and rupture of alveolar septa are common features associated with inflammatory cell infiltration (Huang et al., 2015).

**FIGURE 8:**
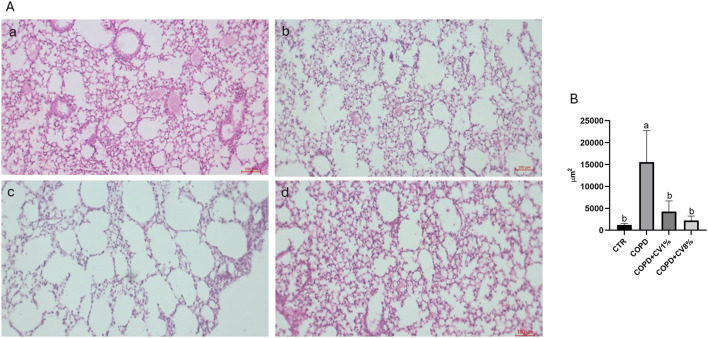
**(A)** Hematoxylin-eosin (H&E) staining of lung tissue from CTR (a), COPD (b), COPD + 1% *C*. vulgaris (c), and COPD + 8% *C*. vulgaris (d) mice. Magnification: bar = 100 µm **(B)**. Calculation of the average alveolar areas from CTR, COPD, COPD+1% C. vulgaris and COPD+ 8% *C*. vulgaris images. CV: *C*. vulgaris. The values are expressed as means ± SD and reported as µm². Different letters (a,b) indicate significant differences among the groups of mice according to the one-way ANOVA test followed by Barlett’s post-test (p ≤ 0.05).

Histological evaluation revealed distinct morphological differences among the experimental groups (Figure 8A). In COPD animals (panel b), enlarged alveoli and disrupted alveolar septa were readily apparent, contrasting with the control group (panel a), which displayed smaller alveoli with intact septal structures. These qualitative observations were supported by morphometric analysis shown in Figure 8B, where the alveolar area was significantly increased in the COPD group compared to healthy controls. Dietary supplementation with *C. vulgaris* led to structural improvements in lung morphology. In the group treated with 1% *C. vulgaris* (panel c), alveolar size was reduced compared to the COPD group but remained slightly larger than in controls. The 8% supplementation group (panel d) showed further improvement, with alveolar dimensions and septal integrity appearing more similar to those of the control group. Quantitative data confirmed that both 1% and 8% *C. vulgaris* treatments resulted in a statistically significant reduction in alveolar area compared to the COPD group, with values not significantly different from the control group (Figure 8B).

#### 3.4.4 Effect of *Chlorella vulgaris* on the relative gene expression of TGF-β

**FIGURE 9:**
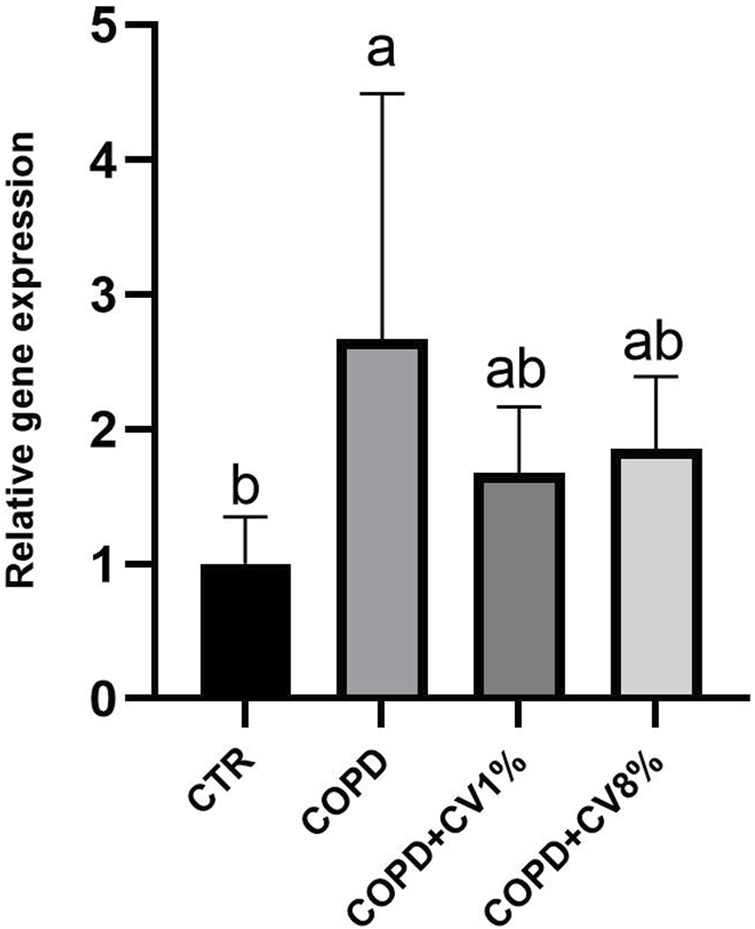
Relative gene expression of TGF-β in mice lungs of all mice from CTR, COPD, COPD+ 1% *C*. vulgaris and 8% *C*. vulgaris. CV: *C*. vulgaris. The values are expressed as means ± SD. Results are normalized for the levels of housekeeping gene β-actin, GAPDH and B2M and referred to the mean of the controls, to which a value of 1 was assigned. Different letters (a, b) indicate significant differences among the groups of mice according to one-way ANOVA test followed by Barlett’s post-test (p ≤ 0.05).

The gene expression levels of TGF-β were analyzed across the four experimental groups to assess the impact of *C. vulgaris* supplementation on fibrotic signaling pathways (Figure 9). The control group exhibited the lowest TGF-β expression, while the COPD group showed the highest levels. Both 1% and 8% *C. vulgaris*-supplemented groups demonstrated a decrease in TGF-β expression relative to the COPD group; however, the reduction was not statistically significant.

#### 3.4.5 Oxidative stress status in the lungs

**FIGURE 10:**
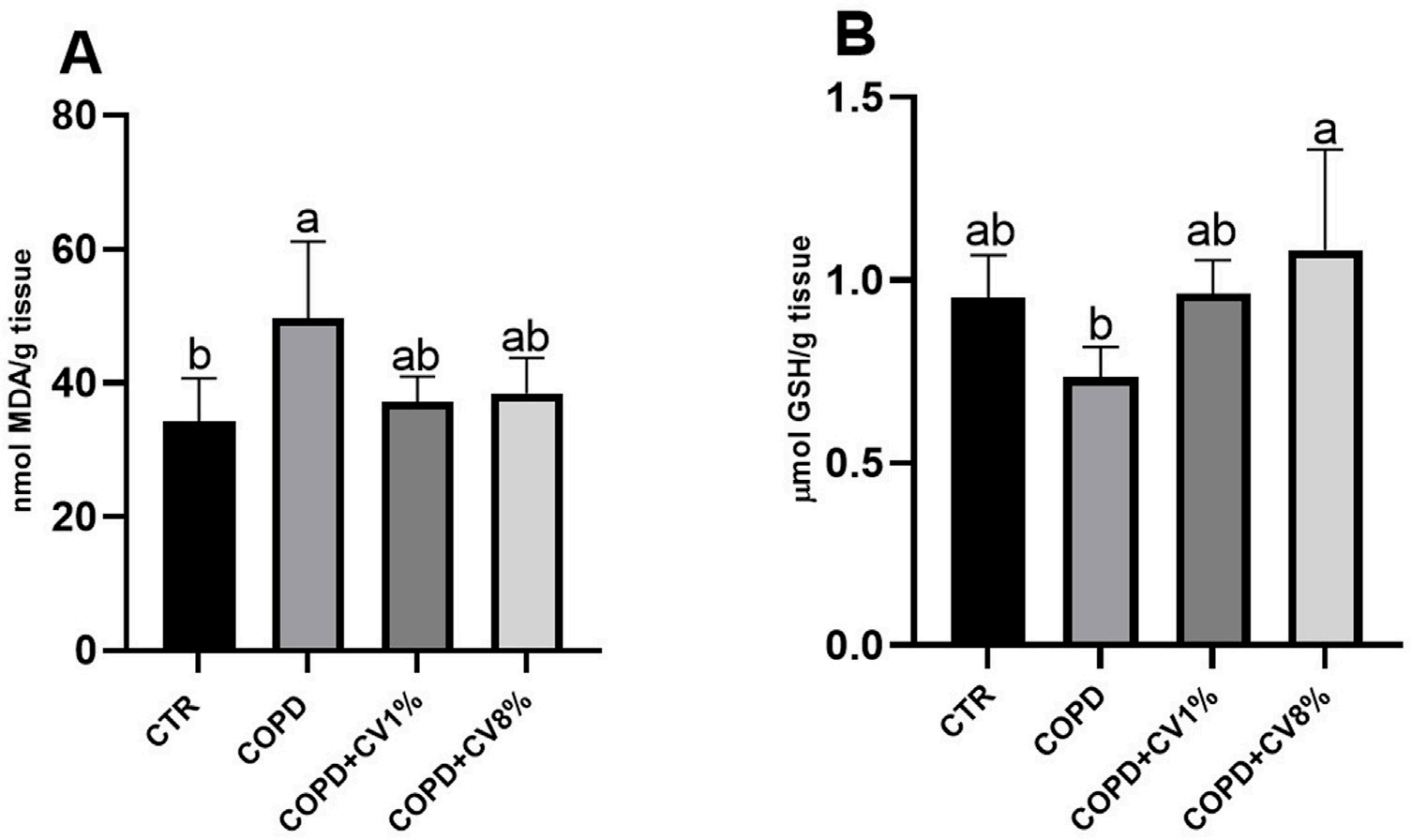
MDA **(A)** and GSH content **(B)** in the lungs of all CTR, COPD, COPD+1% *C*. vulgaris and COPD+8% *C*. vulgaris mice. CV: *C*. vulgaris. The values are expressed as means (n=3) ± SD and reported as nmol MDA/g tissue for MDA and µmol GSH/ g tissue for GSH. Different letters (a, b) indicate significant differences among the groups according to one-way ANOVA test followed by Barlett’s post-test (p ≤ 0.05).

To assess the oxidative status of the lungs, two key biomarkers of oxidative stress were quantified: MDA and GSH. As shown in Figure 10A, MDA levels, an indicator of lipid peroxidation, were significantly elevated in the COPD group compared to the control group, indicating increased oxidative stress associated with disease induction. In the groups treated with 1% and 8% *C. vulgaris*, MDA levels were reduced relative to the COPD group, although the decrease did not reach statistical significance. GSH content, a critical endogenous antioxidant involved in detoxification of reactive oxygen species, was significantly decreased in the COPD group compared to controls (Figure 10B). However, in both groups supplemented with *C. vulgaris*, GSH levels were restored to values comparable to the control group, suggesting a normalization of antioxidant defense mechanisms despite disease induction.

#### 3.4.6 Pearson’s correlation and PCA

**FIGURE 11:**
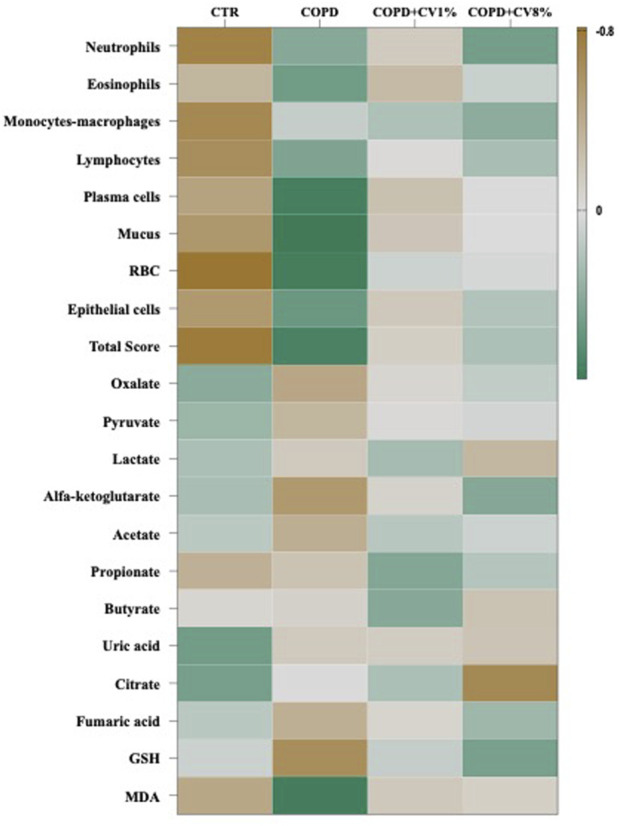
Heat map reflecting Pearson’s correlation coefficients between experimental groups (n = 8) and some parameters analysed during the study, specifically BALF scoring, metabolites of caecal content and markers of oxidative stress status in the lungs. Green colour represents a negative correlation, and brown represents a positive correlation.

To investigate the relationships between treatment groups and various biological parameters, Pearson’s correlation analysis was conducted among the four experimental groups: CTR, COPD, COPD+1% *C. vulgaris*, and COPD+8% *C. vulgaris*. The correlation matrix is visualized in Figure 11. As expected, the CTR group showed strong negative correlations with multiple BALF inflammatory cell types, including neutrophils (r = −0.732), monocytes/macrophages (r = −0.681), lymphocytes (r = −0.643), plasma cells (r = −0.473), mucus (r = −0.562), red blood cells (RBC; r = −0.816), epithelial cells (r = −0.553), and the total inflammation score (r = −0.785). Additionally, negative correlation was observed with the oxidative stress marker malondialdehyde (MDA; r = −0.436), and positive correlations were found with short-chain fatty acids (SCFAs), particularly uric acid (r = 0.492) and citrate (r = 0.471). Conversely, the COPD group demonstrated positive correlations with inflammatory cell types including neutrophils (r = 0.397), eosinophils (r = 0.497), lymphocytes (r = 0.430), plasma cells (r = 0.713), mucus (r = 0.749), RBC (r = 0.719), epithelial cells (r = 0.526), and the total score (r = 0.680). A strong positive correlation with MDA (r = 0.716) and negative correlations with several metabolites, oxalate (r = −0.446), α-ketoglutarate (r = −0.552), and acetate (r = −0.383), were also observed. The COPD+1% *C. vulgaris* group was positively correlated with SCFAs such as propionate (r = 0.412) and butyrate (r = 0.396), suggesting beneficial metabolic effects. The COPD+8% *C. vulgaris* group showed positive correlations with neutrophils (r = 0.478), α-ketoglutarate (r = 0.404), and GSH (r = 0.461), while being negatively correlated with citrate (r = −0.676).

**FIGURE 12:**
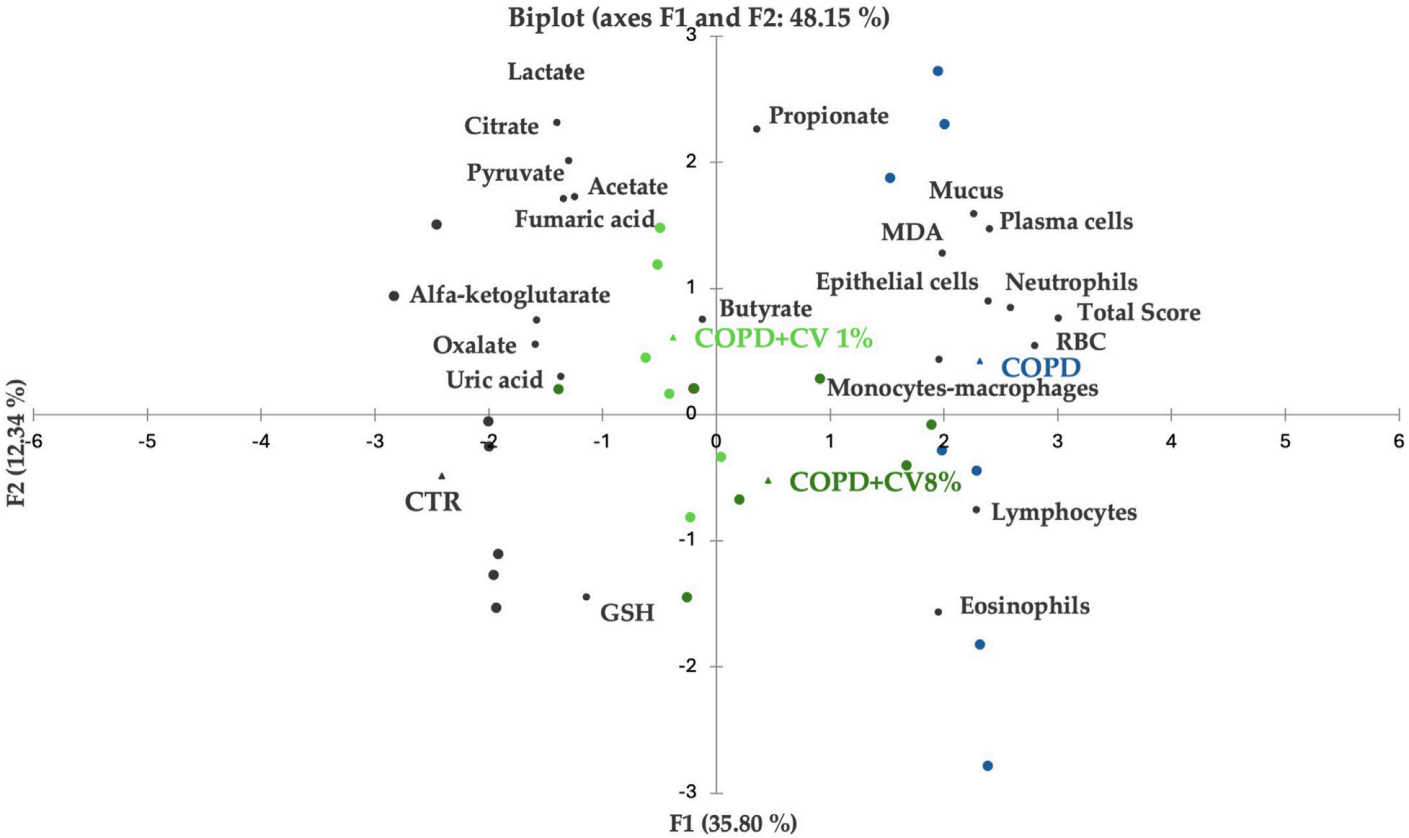
Principal Component Analysis (PCA) of effect of the groups treatment on BALF scoring (neutrophils, eosinophils, monocytes-macrophages, lymphocytes, plasma cells, epithelial cells, mucus, RBC, and total score), caecal SCFA (lactate, acetate, fumaric acid, pyruvate, butyrate, alpha-ketoglutarate, uric acid, oxalate and citrate) and markers of oxidative stress in lung (GSH and MDA): observation score plot (A) and biplot (B) of variables of the first two principal components (F1 and F2). Groups are identified as follows: CTR in black, COPD in grey, COPD+CV1% in light green and COPD+CV8% in dark green. RBC, red blood cells; GSH, glutathione; MDA, malondialdehyde.

PCA was performed to further elucidate the relationships between treatment groups and measured parameters. As shown in Figure 12, the first two components, F1 and F2, explained 13.82% and 36.35% of the variance, respectively (total 48.15%). The analysis revealed four clearly distinct clusters corresponding to each treatment group: CTR, COPD, COPD+1% *C. vulgaris*, and COPD+8% *C. vulgaris*.

## 4 Discussion

When compared to other green microalgae, it is important to consider that the antioxidant profile is highly dependent on variables such as cultivation conditions, species type, and the extraction solvent used (Goiris et al., 2012; López et al., 2011). Abdel-Karim et al. demonstrated that the choice of solvent significantly influences phenolic yield in *C. vulgaris*, with total phenolic content ranging from 0.65 to 3.17 mg GAE/g dw. Their study showed the highest yields with acetone and the lowest with ethanol (Abdel-Karim et al., 2019). The phenolic content observed in our study (1.08 ± 0.01 mg GAE/g dw) is lower than that reported by Maadane et al., who obtained 8.1 ± 0.16 mg GAE/g dw using a similar methodology (Maadane et al., 2015). Nevertheless, our results align with those of Goiris et al., who reported an average polyphenol content of 2.11 mg GAE/g dw across 32 microalgal species (Goiris et al., 2012), and with Dammar et al., who found 0.87 mg GAE/g dw in *C. vulgaris* (Dammar et al., 2023).

The carotenoid concentration in our extract (225.86 ± 2.37 mg/g dw) exceeds values reported by Goiris et al., who observed lower levels across various algal species (Goiris et al., 2012), and by Haoujar et al., who documented values between 3.34 and 5.66 mg/g in four marine strains isolated from M’diq Bay, Morocco (Haoujar et al., 2019). Given the recognized antioxidant properties of carotenoids, these findings underscore the potential of *C. vulgaris* as a source of bioactive pigments. Regarding antioxidant activity, the ORAC value observed is consistent with that of Agregán et al. (29.35 μmol TE/g dw) (Agregán et al., 2018), while the DPPH activity aligns closely with that reported by Pradhan et al. (2.82 ± 0.30 μg TE/g dw), who also highlighted its superior performance compared to ascorbic acid (Pradhan et al., 2021). The FRAP assay revealed a high reducing power (652.77 ± 22.27 mg TE/g dw), in contrast to the lower values reported by Hajimahmoodi and collaborators (Hajimahmoodi et al., 2010). The elevated FRAP values observed in our study may, at least in part, reflect the contribution of carotenoids and polyphenols, which are known to possess strong reducing properties. However, while such compounds could explain the trend, a direct correlation between FRAP results and the quantified levels of polyphenols and carotenoids cannot be assumed without specific correlation analyses. Overall, these results confirm that *C. vulgaris* is rich in antioxidant metabolites and pigments, supporting its potential for application in *in vivo* studies targeting health-promoting properties.

Despite the absence of previous reports on the phenolic profile of *C. vulgaris*, comparisons with other microalgae species highlight the relevance of our findings. For instance, quercetin was the major flavonol in our extract (241.16 μg/100 g dw), surpassing concentrations found in *Nannochloropsis gaditana* (33.4 μg/g dw) and *Scenedesmus* species (551.9 μg/g dw in ethanol/water extract; 884.5 μg/g dw in ethyl acetate extract) (Bulut et al., 2019). Quercetin is known for its potent antioxidant activity, attributed to its hydroxyl groups and catechol-type B-ring, and has demonstrated anti-inflammatory, anticancer, antidiabetic, and antimicrobial effects (Ozgen et al., 2016; Azeem et al., 2023). Notably, Nabavi et al. (2024) reported that quercetin protects the liver from sodium fluoride-induced oxidative stress in rats. Similarly, Zou et al. (2015) showed its efficacy in preventing liver injury caused by perfluorooctanoic acid through oxidative stress and inflammation reduction. Among phenolic acids, gallic acid, vanillic acid, and protocatechuic acid were most abundant. Gallic acid has demonstrated strong antioxidant and anti-inflammatory effects, including suppression of COX-2 and pro-inflammatory chemokines (Locatelli et al., 2013). Protocatechuic acid concentrations in our study were consistent with values observed in *N. gaditana* (21.26 μg/g dw), *Tetraselmis suecica* (40.55 μg/g dw), and *Phaeodactylum tricornutum* (22.83 μg/g dw) (Haoujar et al., 2019). However, Onofrejová et al. (2010) found higher protocatechuic acid and lower vanillic acid in *Spongiochloris spongiosa* (65.7 and 7.3 μg/100 g dw, respectively). Interestingly, Bhuvana et al. (2019) reported citric and caffeic acids as major phenolic acids in *C. vulgaris*, which were found only in low amounts in our extract, underscoring the variability of phenolic profiles due to differences in cultivation and extraction methods. The presence of flavan-3-ols, particularly catechin and epicatechin (110.97 μg/100 g dw), is notable, as no prior studies have reported these compounds in *C. vulgaris*. Catechins exert anti-inflammatory effects by modulating cytokine release, enhancing anti-inflammatory markers, and activating Nrf2 expression (Liang et al., 2011). Oleuropein, also detected in our extract (51.02 μg/100 g dw), is known for its antioxidant and anti-inflammatory properties mediated through cytokine inhibition (Motawea et al., 2020). Verbascoside (13.80 μg/100 g dw), recognized for its antimicrobial and anti-inflammatory effects, particularly against *Staphylococcus aureus*, acts by inhibiting protein synthesis and targeting protein kinase C (Avila et al., 1999). Lastly, smaller quantities of procyanidins (63.07 μg/100 g dw), stilbenoids (6.97 μg/100 g dw), and anthocyanins (6.70 μg/100 g dw) were identified. It is important to note that polyphenol content in microalgae can vary considerably due to differences in species, growth conditions, extraction solvents, and analytical techniques, which likely accounts for the discrepancies between our results and previous studies.

The reduction in plasma AST, ALT, and creatinine levels observed in *C. vulgaris*-supplemented groups suggests not only the absence of toxicity but also a potential improvement in liver and kidney function. This effect may be attributed to the substitution of casein, an animal-derived protein used in the control diet, with *C. vulgaris* as a protein source. The shift in protein composition could have influenced metabolic and detoxification processes, resulting in improved hepatic and renal parameters. These observations align with findings reported by Neumann et al. (2018), who evaluated the safety of diets containing *C. vulgaris*, *Nannochloropsis oceanica*, and *P. tricornutum* in mice and reported no signs of toxicity. Similarly, Elmalawany et al. (2014) demonstrated that oral administration of *Aphanizomenon flos-aquae* at 100 mg/kg for 15 consecutive days did not induce hepatic or renal toxicity. In that study, serum levels of ALT, AST, BUN, and creatinine remained unchanged compared to controls. Although a minor increase in urea and BUN was noted in our study, it did not reach statistical significance and may reflect metabolic adaptations to dietary changes rather than a toxicological response. The more pronounced increase in the 1% group relative to the 8% group may also indicate a dose-dependent normalization effect. Overall, these findings confirm that *C. vulgaris* is safe at the tested concentrations and may even contribute to improved liver and kidney biochemical profiles.

The significant induction of ECOD activity in mice treated with 8% *C. vulgaris* suggests a stimulatory effect on overall cytochrome P450 enzyme function, particularly in pathways involving mixed-function oxidases. Although increases in EROD, AnH, and BqD activities were not statistically significant, the observed trends may reflect a general activation of hepatic detoxification enzymes. Importantly, these enzymatic changes occurred without evidence of hepatotoxicity, as corroborated by the absence of alterations in plasma ALT and AST levels, reinforcing the safety profile of *C. vulgaris* at the tested doses. The results align partially with findings by Kim et al. (2003), who reported a reduction in cytochrome P450 activity following *C. vulgaris* administration in mice with CCl_4_-induced hepatic injury, indicating a possible context-dependent modulatory effect. While increased P450 activity is generally associated with enhanced xenobiotic metabolism, it can, in some cases, result in the formation of reactive intermediates. However, the lack of biochemical markers of liver damage in this study suggests that the enzymatic induction does not translate into adverse effects under the experimental conditions. Nevertheless, at the 8% *C. vulgaris* supplementation level this observed enzymes induction may enhance the clearance of co-administered drugs, potentially reducing their efficacy. Conversely, prolonged induction could also generate higher levels of reactive metabolites, raising safety concerns. While our study did not directly assess pharmacokinetics of specific drugs, these results highlight the importance of considering possible diet-drug interactions when *C. vulgaris* is consumed at higher levels. To further investigate the enzyme activity patterns, we assessed transcriptional changes in selected cytochrome P450 genes. The upregulation of CYP2C29 in response to both 1% and 8% *C. vulgaris* diets supports the notion of gene-level modulation. Interestingly, previous studies have identified CYP2C29 as a key factor in liver homeostasis. In a diabetic steatosis model induced by a high-fat diet and streptozotocin, Vornoli et al. (2014) reported downregulation of the rat homolog CYP2C11, indicating that impaired expression may be linked to hepatic pathology.

Additionally, Wang et al. (2020) showed that mice with hepatocellular carcinoma exhibited decreased CYP2C29 transcription, and that transfection with a CYP2C29 plasmid led to significant reductions in AST and ALT levels, suggesting a hepatoprotective role for this enzyme. Therefore, the overexpression of CYP2C29 observed in our study may represent a compensatory protective mechanism, potentially contributing to improved liver function and resistance to hepatic damage (Figure 3).

The observed upregulation of CAT and GSR enzymatic activities following *C. vulgaris* supplementation supports its role in enhancing antioxidant defenses. These findings are in agreement with the study by Sikiru et al. (2019), which demonstrated that dietary inclusion of *C. vulgaris* in white rabbits significantly reduced reactive oxygen species and increased total antioxidant concentrations in serum, liver, and uterus. Similar results were reported by Panahi et al. (2012), in which chronic cigarette smokers receiving *C. vulgaris* supplementation for 6 weeks exhibited improved serum antioxidant levels and reduced lipid peroxidation. Our data are also supported by findings from Napolitano et al. (2020), in which supplementation with *Chlorella sorokiniana*, another *Chlorella* species, led to decreased levels of hydrogen peroxide and oxidized glutathione by increasing CAT and GSR activities. The consistency between enzyme activity and gene expression of CAT and GSR in our study suggests a genuine upregulation of these components of the antioxidant defense system by *C. vulgaris*. In contrast, the slight increase in DT-diaphorase activity observed in the 8% group was not mirrored at the transcriptional level, indicating that *C. vulgaris* supplementation at both concentrations was insufficient to significantly activate the Nrf2 pathway. This transcription factor regulates genes encoding detoxifying enzymes, such as DT-diaphorase and heme oxygenase-1 (HO-1), in response to oxidative stress (Napolitano et al., 2020). The lack of Nrf2 activation in our study may be attributed to low oxidative stress conditions, as also observed in Napolitano et al. (2020), where animals fed *C. sorokiniana* showed reduced ROS production and consequently no Nrf2 activation. Further supporting the antioxidant potential of *C. vulgaris*, Firouzeh et al. (2024) demonstrated that quercetin, the predominant flavonoid in our extract, enhanced the activity of several antioxidant enzymes, particularly catalase, in a diabetic rat model. Similarly, Świderska-Kołacz et al. (2021) reported that dietary supplementation with *Pinnularia borealis*, an alga rich in carotenoids like *C. vulgaris*, led to a significant increase in GSR activity across all tested concentrations. Moreover, oleuropein, another major phenolic component of *C. vulgaris*, has shown potent antioxidant effects. In a rat model of oxidative stress induced by carbon tetrachloride (CCl_4_), treatment with oleuropein (300 mg/kg) significantly increased the activities of SOD, GSH, and CAT, thereby restoring enzyme function and conferring protection against hepatic damage (Buzdar et al., 2024). Lastly, phenolic compounds from *Spirulina platensis* demonstrated similar antioxidant effects in diabetic rats, where treatment reduced malondialdehyde (MDA) levels and increased catalase activity. The authors attributed these effects to key phenolic components, including vanillic acid, which was also identified in *C. vulgaris* in our study at comparable levels (Agregán et al., 2018).

Our analysis of BALF cellular composition confirms the inflammatory nature of COPD and highlights the modulatory effects of *C. vulgaris* supplementation. The significant increase in neutrophils, lymphocytes, epithelial cells, and mucus in the COPD group mirrors previous findings reported by Rodrigues et al. (2021), which identified inflammatory cell infiltration as a hallmark of COPD pathology. Notably, 1% *C. vulgaris* supplementation significantly reduced neutrophil counts, mucus production, and overall inflammatory scores, suggesting an anti-inflammatory effect at this concentration. This is in line with the study by Singanayagam et al. (2015), where COPD was induced using porcine pancreatic elastase (PPE) and lipopolysaccharide (LPS), and subsequent BALF analysis revealed elevated neutrophils and mucus, patterns similarly reversed by effective treatments. Our findings are also consistent with the broader literature on respiratory inflammatory diseases. For example, Bottoms et al. (2010) demonstrated that asthmatic mice exhibited increased BALF levels of monocytes/macrophages, eosinophils, lymphocytes, and neutrophils, indicating that COPD and asthma share overlapping inflammatory signatures. Interestingly, the dose-dependent effects observed in BALF, with 1% *C. vulgaris* more effective on neutrophils and 8% more effective on eosinophils, suggest differential immunomodulatory actions. Lower supplementation may primarily attenuate acute neutrophilic inflammation, while higher levels could favor modulation of eosinophil-driven responses, possibly via shifts in oxidative balance, cytokine signaling, or gut-lung axis-mediated immune regulation. Furthermore, the current results align with emerging evidence supporting the anti-inflammatory properties of microalgae and their bioactive components. Phycocyanin, a compound found in certain microalgae, has been demonstrated to modulate inflammatory pathways (Li et al., 2024). Our study also highlights the relevance of quercetin, the most abundant phenolic compound identified in our *C. vulgaris* extract. In an acute lung injury (ALI) model induced by LPS, Huang et al. (2015) found that quercetin significantly reduced neutrophil infiltration in BALF, an effect mirrored in our 1% *C. vulgaris* group. Additionally, gallic acid, another phenolic compound present in *C. vulgaris*, has demonstrated protective effects in a mouse model of COPD induced by PPE and LPS. In this model, Singla et al. (2021) reported that gallic acid significantly reduced neutrophil counts in BALF, supporting its therapeutic potential. These studies collectively reinforce our findings that *C. vulgaris* supplementation, particularly at 1%, can attenuate the pulmonary inflammatory response associated with COPD. While 8% supplementation exhibited partial effects (e.g., reduced eosinophils and RBCs), it did not significantly improve total inflammatory scores or neutrophil infiltration, suggesting a potential dose-response plateau or biphasic effect. Further studies may be warranted to clarify these differential responses and to optimize the therapeutic dosage.

Histopathological evaluation confirmed the structural lung damage characteristic of COPD, including alveolar enlargement and septal rupture. These findings are consistent with those of Singanayagam et al. (2015), who demonstrated significant histological damage in a murine model of COPD induced by PPE and LPS, followed by RV infection. Our results demonstrated that dietary supplementation with *C. vulgaris*, particularly at 8%, mitigated the histological signs of emphysema. The observed reduction in alveolar size and the preservation of septal integrity suggests that *C. vulgaris* exerts a protective effect on lung architecture. These results contrast with findings by Panahi et al. (2012), who reported no morphological improvements following administration of *C. vulgaris* extract in a similar model. Such discrepancies may be attributed to differences in experimental design, dosage, duration of treatment, or bioactive compound availability in the extract. The beneficial effects of *C. vulgaris* on lung morphology are likely attributable to its high content of carotenoids and polyphenols, particularly quercetin. Carotenoids have well-documented antioxidant and anti-inflammatory properties that help prevent emphysematous damage and chronic bronchitis by reducing oxidative stress (Islam et al., 2022). Similarly, quercetin has been shown to exert protective effects in various COPD models. Farazuddin et al. (2018) reported that quercetin administration in mice infected with RV significantly reduced alveolar damage, aligning with our findings. Huang et al. (2015) also demonstrated a significant reduction in lung injury in rats pretreated with quercetin, further supporting the hypothesis that this compound contributes to the amelioration of COPD pathology. In summary, our histopathological findings support the hypothesis that dietary *C. vulgaris*, through its rich content of carotenoids and quercetin, may offer a protective effect against COPD-associated structural lung damage. The observed improvements in alveolar morphology suggest that such supplementation could represent a viable strategy to mitigate disease progression, largely due to its anti-inflammatory and antioxidant mechanisms of action.

TGF-β is a key cytokine involved in inflammation, tissue remodeling, and the promotion of fibrosis, an exacerbating feature of COPD that contributes to progressive deterioration of pulmonary architecture. The elevated TGF-β expression observed in the COPD group is consistent with its established role in collagen deposition and fibrotic tissue remodeling (Xu et al., 2024). These findings align with previous evidence indicating that COPD pathogenesis includes heightened fibrogenic signaling. Comparable results have been reported in models of LPS-induced lung injury, where increased TGF-β expression was detected in both lung tissue and BALF, alongside elevated levels of other pro-fibrogenic mediators such as IL-17 and MMP-9 (Wang et al., 2021). These observations support the idea that the upregulation of TGF-β may serve as a molecular correlate of fibrotic remodeling in COPD. Although the observed reductions in TGF-β expression in the *C. vulgaris*-supplemented groups were not statistically significant, the downward trend is consistent with the known anti-inflammatory and anti-fibrotic properties of the bioactive compounds contained in this microalga. Prior studies have identified carotenoids, abundant in *C. vulgaris*, as key modulators of pulmonary inflammation, capable of attenuating inflammatory cell infiltration and downregulating fibrogenic mediators including TGF-β, TNF-α, IL-6, and IL-1β (Cichoński and Chrzanowski, 2022; Islam et al., 2022). In addition to carotenoids, polyphenolic compounds such as catechins have also demonstrated the ability to reduce TGF-β expression in murine models of lung inflammation (Liang et al., 2011), lending further support to the potential therapeutic role of *C. vulgaris* in mitigating COPD-associated fibrosis. While the observed reduction in TGF-β gene expression following *C. vulgaris* supplementation did not reach statistical significance, the data suggest a possible modulatory effect of its bioactive components on fibrotic pathways in COPD. These findings warrant further investigation with extended treatment durations or higher sample sizes to conclusively determine the therapeutic potential of *C. vulgaris* in preventing fibrotic progression in chronic lung disease.

Oxidative stress plays a central role in the pathophysiology of COPD, contributing to airway inflammation, tissue remodeling, and alveolar damage. In this study, the COPD group exhibited elevated MDA levels and reduced GSH concentrations in lung tissue, consistent with the presence of enhanced lipid peroxidation and compromised antioxidant defenses. These findings are in agreement with clinical observations in COPD patients, where increased MDA and reduced antioxidant enzyme activity, including glutathione peroxidase, have been reported (Vibhuti et al., 2007). Although the reduction in MDA levels following *C. vulgaris* supplementation did not achieve statistical significance, the downward trend suggests a potential mitigating effect on oxidative stress. More notably, GSH levels were fully restored in both microalgae-treated groups, indicating a beneficial impact on endogenous antioxidant capacity. These results support the hypothesis that *C. vulgaris* may exert protective effects against oxidative injury in lungs through enhancement of redox balance. Clinical data corroborate these findings. Panahi et al. (2012) reported that administration of *C. vulgaris* extract in patients with asthma or COPD led to a significant reduction in MDA and an increase in GSH, suggesting a systemic antioxidant effect of the microalga in chronic respiratory conditions. Similarly, Chaudhari and Baviskar (2021) demonstrated that *C. vulgaris* supplementation in a murine model of acute inflammation (paw edema) resulted in decreased MDA and increased GSH levels, reinforcing its role in oxidative stress modulation. The previously observed increases CAT and GSR enzymatic activities with algal integration support enhanced antioxidant defense, which is particularly relevant in the COPD model. Upregulation of these enzymes may reduce oxidative stress and mitigate reactive oxygen species-driven tissue injury, thereby contributing to the actually observed amelioration of COPD-associated pathology. The antioxidant activity of *C. vulgaris* is primarily attributed to its rich content of bioactive compounds such as carotenoids, polyphenols, and vitamins, which are known to scavenge free radicals and upregulate antioxidant defenses. The oxidative stress reduction observed in this study likely contributes to the improved histopathological outcomes and reduced inflammatory cell infiltration seen in BALF, further supporting the therapeutic potential of *C. vulgaris* in COPD management.

The correlation analysis and PCA results provide a comprehensive overview of the systemic and pulmonary responses to COPD and its dietary modulation through *C. vulgaris* supplementation. The negative correlations observed between the CTR group and BALF inflammatory markers, as well as MDA, underscore the absence of inflammation and oxidative stress in healthy animals. This group’s positive correlation with several caecal SCFAs, including uric acid and citrate, supports a healthy metabolic profile, which may reflect a balanced gut-lung axis. In contrast, the COPD group exhibited a pro-inflammatory and pro-oxidative profile, characterized by elevated BALF immune cell infiltration and increased MDA levels. These results are consistent with the pathophysiological features of COPD, where oxidative stress and immune dysregulation drive tissue damage. Moreover, the negative correlations with gut-derived metabolites such as acetate and α-ketoglutarate suggest microbial and metabolic dysbiosis, a growing area of interest in the gut-lung axis in COPD pathogenesis (Kotlyarov, 2022). Dietary supplementation with *C. vulgaris*, particularly at 1%, was associated with metabolic patterns closer to the control group. The COPD+1% group clustered with several beneficial caecal metabolites (lactate, acetate, fumaric acid, pyruvate, butyrate, α-ketoglutarate, and uric acid), suggesting that *C. vulgaris* modulates the gut microbiota to enhance SCFA production. These SCFAs are known to have immunomodulatory effects, potentially contributing to reduced pulmonary inflammation through gut-lung crosstalk. Interestingly, the 8% *C. vulgaris* group showed a distinct profile, characterized by a positive correlation with neutrophils and GSH, and a negative correlation with citrate. While this group did not cluster as closely with the control group as the 1% group did, its proximity in the PCA plot still indicated partial restoration of a healthy phenotype. The PCA effectively distinguished all four groups based on combined inflammatory, oxidative, and metabolic data, demonstrating the potential of multivariate analysis in elucidating complex biological responses. Notably, both *C. vulgaris* supplementation groups were more closely aligned with the CTR group than with the COPD group, reinforcing the therapeutic potential of microalgal supplementation in mitigating COPD-induced changes.

Our findings highlight the capacity of *C. vulgaris* to improve systemic and pulmonary parameters in a murine COPD model, likely through its modulation of oxidative stress, inflammatory responses, and gut-derived metabolites. This study is the first to evaluate the effects of *C*. *vulgaris* extracts in a COPD experimental model. The detailed phenolic characterization and the observed dose-dependent differences in antioxidant and anti-inflammatory activities provide novel insights into the biological potential of *C. vulgaris*.

## 5 Conclusion

The extract of *C. vulgaris* contained significant phenolic compounds, predominantly flavonols with quercetin as the main representative, along with phenolic acids such as gallic, vanillic, and protocatechuic acids. Carotenoids were the most abundant pigments and overall bioactive compounds. Toxicological evaluation revealed that dietary supplementation with *C. vulgaris* increased hepatic CYP2C29 gene expression and ECOD activity, suggesting a protective liver effect, while other isoforms remained unchanged. Blood analyses confirmed the absence of liver or kidney toxicity, with evidence of improved organ health at both doses. Antioxidant enzyme activity and gene expression of glutathione reductase and catalase were enhanced by supplementation. In a COPD murine model, *C. vulgaris* demonstrated anti-inflammatory and antioxidant effects: BALF analysis showed reduced inflammatory cell infiltration (neutrophils, eosinophils, plasma cells), decreased mucus production, and epithelial infiltration. Oxidative stress markers improved, with increased GSH and reduced MDA levels, correlating with histopathological improvements such as reduced alveolar damage. Caecal SCFA analysis indicated beneficial gut microbiota and metabolic modulation, especially with 1% supplementation. Collectively, these results highlight *C. vulgaris*’ potential as a therapeutic agent for COPD by modulating inflammation, oxidative stress, and intestinal health, likely through synergistic actions of its abundant secondary metabolites, particularly quercetin, gallic acid, vanillic acid, catechins, epicatechins, oleuropein, verbascoside, and carotenoids, conferring pronounced anti-inflammatory and antioxidant effects.

## Data Availability

The original contributions presented in the study are included in the article/Supplementary Material, further inquiries can be directed to the corresponding author.
