# Supplementary material for: Toxicological assessment of Chlorella vulgaris and its potential preventive effect in a chronic obstructive pulmonary disease (COPD) mouse model
Source: Front Toxicol. 2025 Nov 11;7:1654583. doi: 10.3389/ftox.2025.1654583 (PMC12643852; doi:10.3389/ftox.2025.1654583)
Supplement: Supplementary file 1 [file DataSheet1.docx]

Supplementary Material

1. Evaluation of caecal metabolomic changes

To prepare the caecal samples, approximately 45 mg of caecal content was thawed and dissolved in 500 µL of ultrapure bidistilled water (ELGA Ultrapure Laboratory Water). The mixture was homogenized using vortexing and sonication (40 kHz for 5 minutes), followed by centrifugation at 14,000 rpm for 10 minutes to isolate the supernatant. The supernatant was then deproteinized using Microcon® centrifugal filters with a 3 kDa cutoff (Merk, Milan, Italy) (named 3 kDa filtered samples). These were diluted threefold with 5 mM sulfuric acid, filtered through a Mini-Uniprep RC 0.20 µm filter (Agilent Technologies, Milan, Italy), and prepared for HPLC analysis. The analysis was performed on an Agilent 1260 Infinity HPLC system (Agilent Technologies, Santa Clara, CA, USA) equipped with a quaternary pump (G1311B), a high-performance degasser, a TCC G1316A thermostat, a 1260ALS autosampler (G1329B), and a UV/vis diode array detector (1260 DAD G4212B). Identification of short-chain fatty acids (SCFAs) was achieved by comparing retention times and UV spectra with standard compounds, using a detection wavelength of 220 nm to minimize interference from high-absorbing compounds. Chromatographic separation utilized a Zorbax Phenyl-Hexyl RP C18 column (250 × 4.6 mm, 4 µm particle size) maintained at 45 °C. The elution protocol included 15 minutes of isocratic elution with 0.1% phosphoric acid, a 10-minute gradient to 80% methanol, and a final 10-minute isocratic phase in 80% methanol. The column was rinsed with 100% methanol for 15 minutes and re-equilibrated. All solutions used in the preparation and analysis were filtered through 0.22 µm regenerated cellulose membranes (Millipore, Milan, Italy) to ensure sample purity.

1.1 Modulation of caecal metabolites due to the administration of *Chlorella vulgaris* in COPD disease

Supplementary Figure 1 shown the caecal content of mice in terms of short-chain fatty acids. In particular there are no statistically significant differences between the four animal groups regarding the levels of lactate, acetate, uric acid, fumaric acid, and butyrate. Regarding the propionate content, a statistically significant increase was recorded at the concentration of 1% *C. vulgaris* compared to the control, with a slight increase also observed in the presence of COPD and COPD+CV8%. The content of oxalate and pyruvate showed similar changes; as seen in the figure, the levels of these two fatty acids were significantly reduced in the presence of COPD, and then increased again, reaching values statistically similar to the control with the two concentrations of *C. vulgaris.* A similar trend was observed for alpha-ketoglutarate as with the two previously mentioned fatty acids, except for the treatment with CV1%, where a slightly significant increase was recorded compared to the group with the disease. The citrate content remained unchanged, except for the COPD+CV8% group, where a statistically significant reduction was observed.

The results of our study and those of Li *et al*. show both similarities and differences in SCFAs levels in COPD patients. Both studies highlight a significant reduction in total SCFAs levels in COPD patients, but in our case, no significant differences were found for acetic acid, whereas in Li *et al*.'s study, this SCFA was significantly reduced. Furthermore, both studies agree in not observing significant differences in other SCFAs such as propionic and butyric acids, suggesting that the progression of the disease has a lesser impact on these fatty acids (Li et al., 2021). Another difference lies in the study population: in Li *et al*.'s study, patients were divided by disease stage, whereas in our study, no such stratification was made. These results suggest that intestinal dysbiosis and SCFAs production may vary with the severity of COPD, but also that differences between the two studies could be attributed to the population composition and the methodological approaches used.

**Supplementary Figure 1.** Caecal content of all CTR (
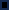
), COPD (
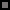
), COPD+CV1% (
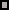
) and COPD+CV8% (
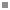
) mice. Values are expressed as means ± SD and reported in mM. For each fatty acid, the presence of different letters (a, b, c) indicates significant differences among groups according to the one-way ANOVA- test followed by Barlett’s test post-test (p ≤ 0.05).

2. References

Li, N., Dai, Z., Wang, Z., Deng, Z., Zhang, J., Pu, J., Cao, W., Pan, T., Zhou, Y., Li, J., Li, B., and Ran, P. (2021). Gut microbiota dysbiosis contributes to the development of chronic obstructive pulmonary disease. *Respir Res* **22**, 274. doi: 10.1186/s12931-021-01872-z
